# A 21st Century View of Allowed and Forbidden Electrocyclic Reactions

**DOI:** 10.1021/acs.joc.3c02103

**Published:** 2023-12-28

**Authors:** Qingyang Zhou, Garrett Kukier, Igor Gordiy, Roald Hoffmann, Jeffrey I. Seeman, K. N. Houk

**Affiliations:** † Department of Chemistry and Biochemistry, University of California, Los Angeles, California90095, United States; ‡ Department of Chemistry and Chemical Biology, Cornell University, Ithaca, New York14850, United States; § Department of Chemistry, University of Richmond, Richmond, Virginia 23173United States; ∥ Department of Chemistry and Biochemistry, University of California, Los Angeles, California90095-1569. United States

## Abstract

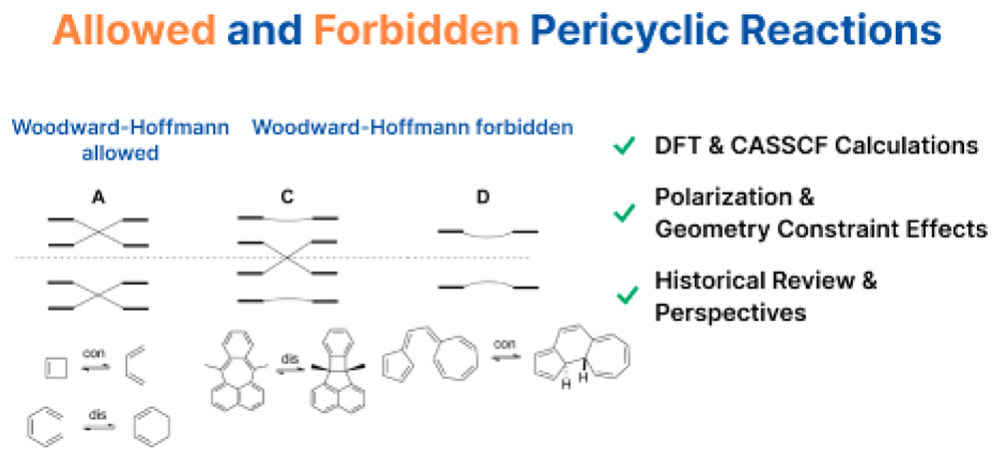

In 1965, Woodward and Hoffmann proposed a theory to predict the stereochemistry of electrocyclic reactions, which, after expansion and generalization, became known as the Woodward–Hoffmann Rules. Subsequently, Longuet-Higgins and Abrahamson used correlation diagrams to propose that the stereoselectivity of electrocyclizations could be explained by the correlation of reactant and product orbitals with the same symmetry. Immediately thereafter, Hoffmann and Woodward applied correlation diagrams to explain the mechanism of cycloadditions. We describe these discoveries and their evolution. We now report an investigation of various electrocyclic reactions using DFT and CASSCF. We track the frontier molecular orbitals along the intrinsic reaction coordinate and modeled trajectories and examine the correlation between HOMO and LUMO for thermally forbidden systems. We also investigate the electrocyclizations of several highly polarized systems for which the Houk group had predicted that donor–acceptor substitution can induce zwitterionic character, thereby providing low-energy pathways for formally forbidden reactions. We conclude with perspectives on the field of pericyclic reactions, including a refinement as the meaning of Woodward and Hoffmann’s “Violations. There are none!” Lastly, we comment on the burgeoning influence of computations on all fields of chemistry.

## 1 Introduction

**Figure 1:**
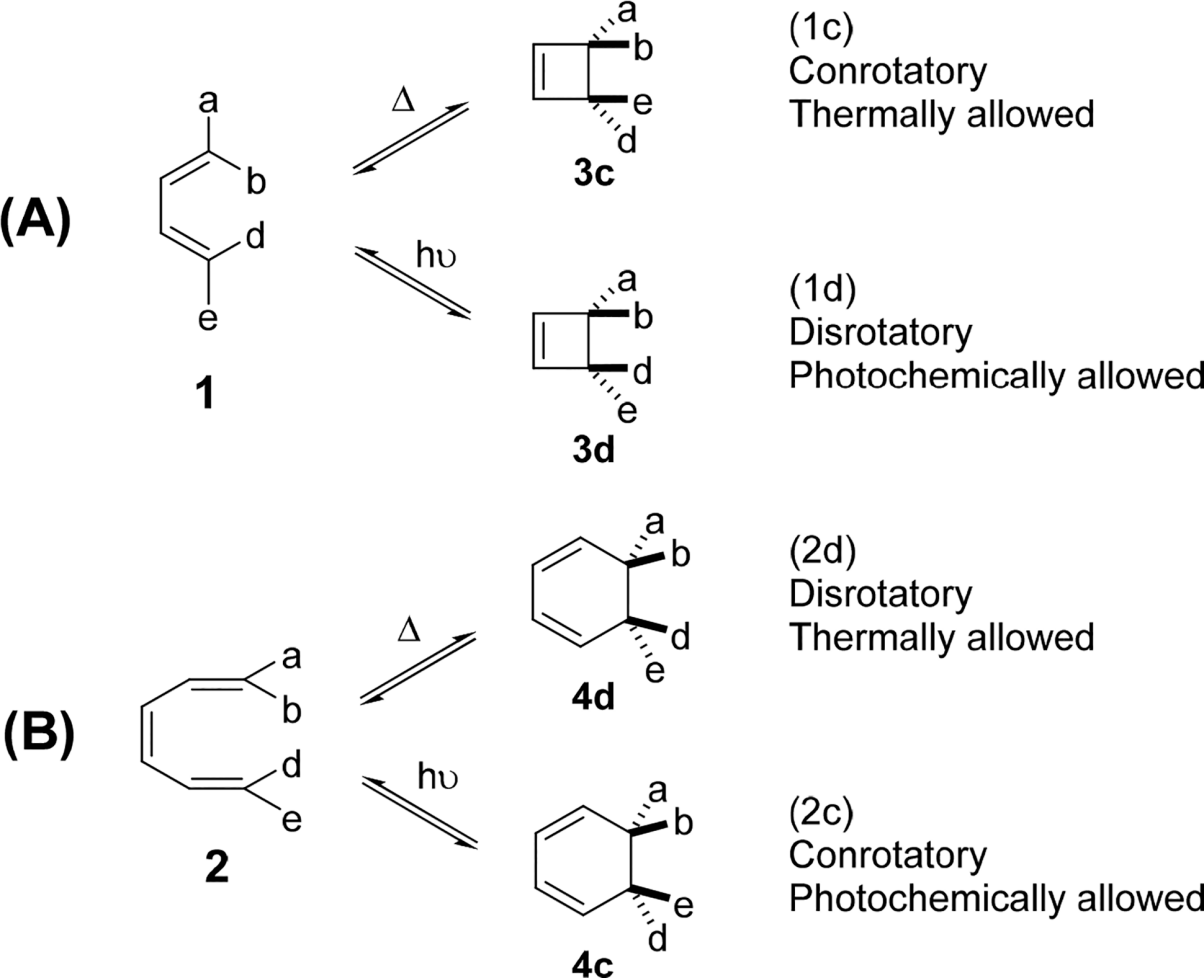
Definition of allowed and forbidden electrocyclizations of substituted 1,3-butadienes (**1**) and 1,3,5-hexatrienes (**2**). (A) Thermal and photochemical four-electron electrocyclizations. (B) Thermal and photochemical six-electron electrocyclizations. When the reaction is thermally allowed, it is photochemically forbidden, and the converse.

The Woodward–Hoffmann (W–H) publications of 1965 and thereafter clearly distinguished between “allowed” and “forbidden” pericyclic reactions.^1−10^ R. B. Woodward and Roald Hoffmann defined “pericyclic reactions” as “reactions in which all first-order changes in bonding relationships take place in concert on a closed curve.”^9,11^ Their first communication in 1965^4^ introduced the idea of electronic control of stereoselectivity of cyclizations that they named electrocyclizations. Examples of allowed and forbidden electrocyclizations for 1,3-butadiene (**1**) and 1,3,5-hexatriene (**2**) are shown in Figure 1.

In further communications in 1965,^1,5,12^ and further explications published in 1967–1969,^7−9,11^ Woodward and Hoffmann proposed a set of “selection rules” that summarized the reactivity and stereochemical consequences of all pericyclic reactions.^9,11^ These selection rules only mention “symmetry-allowed” reactions, implying that other pericyclic or seemingly pericyclic reactions are orbital-symmetry-forbidden and would occur only at higher temperatures or in nonconcerted pathways.

Woodward and Hoffmann began the antepenultimate section of their 1969 treatise with the words,

“Violations

There are none!”^9,11^

Over the years, imaginative chemists have sought, and indeed sometimes claimed, to have found pericyclic allowed reactions that proceed in stereochemically defined pathways that are in violation of the Woodward–Hoffmann selection rules. Indeed, even 60 years later, chemists continue to challenge the validity and generality of the W–H rules,^13−19^ although they are in general use in the experimental and pedagogical communities. Indeed, there are clear and well-known “violations” that occur due to unique structural features that prevent allowed reactions from occurring and only permit the forbidden pathway.^20−22^ Very recently, the Houk group showed how high polarity, even in hydrocarbons, can overcome the restrictions of the Woodward–Hoffmann Rules.^13^

The conclusion section of the Woodward–Hoffmann 1969 summary treatise is more descriptive and fundamental than focusing on violations.^13^ Woodward and Hoffmann wrote,

“We have now explicated the principle of conservation of orbital symmetry and exemplified its use. It may perhaps be appropriate here to emphasize that the central content of the principle lies in the incontrovertible proposition that a chemical reaction will proceed more readily, and more bonding may be maintained throughout the transformation. Consequently, we cannot doubt that the principle will endure, whatever the language in which it may be couched, or whatever greater precision may be developed in its application and extension.”^9,11^

The Woodward–Hoffmann rules did more than “endure.” According to a recent study on the history and philosophy of chemistry, they did more than endure, they precipitated a revolution in chemistry!^23^

Specifically, the W–H rules describe how orbital symmetry influences maximum bonding as maximum bonding implies lower energy. The W–H rules are based upon whether the occupied bonding orbitals are smoothly transformed to those of the product without any symmetry-imposed barrier, i.e., an allowed reaction. In his quantum chemical calculations with Woodward in 1964 and 1965, Hoffmann used extended Hückel theory (eHT)^1−5^ which captured the essential energetic differences between allowed and forbidden reactions.

Woodward and Hoffmann noted that orbital symmetry influences activation barriers of reactions by influencing the orbital energies and thus the potential energy of the reaction. But they did not focus on the molecular orbitals and energetics of forbidden reactions. In this Perspective, one of our goals is to connect the early frontier molecular orbital theory (FMO theory) explanations and eHT calculations of Hoffmann with modern computational analyses. We report modern computations and contrast the qualitative deductions of 1965 with the more quantitative methods available today. We focus on the relationship between potential energy surfaces (PES), correlation diagrams, Walsh diagrams,^24,25^ and intrinsic reaction coordinate (IRC) diagrams. We explore several thermal electrocyclizations that are allowed or forbidden by Woodward and Hoffmann’s selection rules. A key aspect of this Perspective is the examination of the behavior of the HOMOs and LUMOs during the course of W–H forbidden reactions. We asked, is there still continuous bonding through the transition states in forbidden reactions as found in allowed reactions? What can we learn about chemical reactivity by focusing on W–H forbidden reactions rather than W–H allowed reactions? Photochemical electrocyclizations involve more complicated potential energy surfaces^26−30^ and are not discussed in detail here. We also explored the meaning of "violations" of the W-H rules.

## 2 Early Apparent Violations to the Woodward–Hoffmann Rules

From the early days of pericyclic reactions, some stereochemical outcomes in pericyclic reactions could be termed “mixed”, in the sense that the product stereochemistries observed corresponded to a mixture of formally “allowed” and “forbidden” outcomes. Once these were determined not to be experimental artifacts, they became objects of some theoretical interest. The predominant reaction of this type was a sigmatropic shift.

Let us mention some of the early work because it was done by very good experimentalists who also had a sound understanding of the chemical physics involved. The work of John Baldwin and later Phyllis Leber,^15−18^ William von E. Doering,^21,22,31^ Jerome A. Berson,^32−34^ and Barry Carpenter^35−43^ and their groups was prominent here, for several reasons —it was reliable, extensive, and carefully thought through—the fledgling physical organic chemist of the time would have been well advised to study their work. For Barry Carpenter, it led to a lifelong interest, with remarkable results, to which we will return.

To these researchers—and those who took their work a step further, Daniel A. Singleton,^106,107^ Stephen L. Craig,^71,73−75^ Dean J. Tantillo,^108−113^ Charles Doubleday,^44^ and K. N. Houk^114−121^—it was necessary to reach beyond the hill-climbing metaphor of a standard potential energy surface, by then barely 30 years young. One had to make contact with modern chemical physics, get used to multidimensional potential energy surfaces, to the coupling of electronic, vibrational, and rotational energy levels, and finally begin to run ensembles of molecules traversing these surfaces with velocities and momenta.

At the time, the shifting mental landscape of the researchers beginning to think about the outcome of mixed stereochemistries, or, if you prefer, allowed and forbidden stereochemical outcomes coexisting, is replete with varying terminology: dynamic effects, caldera, bifurcations, driven by entropy, continual biradicals. This is best illustrated by an example, the end of the abstract of an important paper by Doering and Sachdev:

“In the dynamic aspects of bond breaking we believe to have found the basis for a consistent conceptual scheme, that of the continuous diradical as the internally rotationally coupled extension of stretching vibrational modes. Incapable of being trapped, the continuous diradical represents families of energetically and orbitally not concerted transition states the stereochemical differences among which originate in energetic preferences among the observationally independent, internally rotational components.”^21^

People did not lose interest in violations. Berson wrote a paper with the title *Orbital-Symmetry-Forbidden Reactions*.^34^ Scholars are thinking deeply about forbidden reactions.

Fifty years later, we may be close to having the language and physics and chemistry and computing power to understand dynamic effects. Not quite yet. But that is NOT the subject of our paper. We look, in orbital detail and with modern quantum chemical methodology, at “classically forbidden” electrocyclizations. As we will show, there are interesting things to learn from this exercise in the theoretical construction of violations.

## 3 Reaction Coordinate Diagrams: 1964 to the Present

When Hoffmann began his collaboration with Woodward in May 1964,^12,45−48^ he studied certain valence isomerizations known as “no-mechanism reactions.”^49−52^ This colloquialism reflects the fact that the reactions have no intermediates or multiple steps, the usual definition of a concerted mechanism. These “no-mechanism reactions” were later named pericyclic reactions^9,11^ by Woodward and Hoffmann. Hoffmann developed a precursor to extended Hückel Theory (eHT) while a graduate student in William Lipscomb’s group at Harvard and in collaboration with other members of that group.^53−55^ Hoffmann improved that theory and began to use the newly named eHT in the early days of his Harvard Junior Fellowship. EHT is essentially Hückel theory extended to include σ orbitals and to numerically compute values of Hückel’s α and β for planar as well as nonplanar molecules, i.e., calculations that could be performed for three-dimensional molecules using a computer program—a state-of-the-art achievement in the early 1960s.^54,56^

In mid-1964, Hoffmann studied the thermal and photochemical valence isomerizations of cyclobutene ⇋ 1,3-butadiene (Figure 1, Eq 1) and 1,3-cyclohexadiene ⇋ 1,3,5-hexatriene (Figure 1, Eq 2).^4,12^ He determined the energies of cyclobutene with its C(3)–C(4) bond elongated to 2.14 Å and with the two terminal methylene groups rotated to model its ring opening using eHT. He also determined the energies of 1,3-butadiene as the terminal methylene groups rotated in what he called the “syn” and the “anti” directions. Hoffmann’s quite simplified, even rudimentary potential energy surface^4^ consisted of seven points!^12^ These calculations may well have been the first PES calculation done on a complex organic chemical reaction. These and related results formed the theoretical basis for the first Woodward–Hoffmann publication.^4^

**Figure 2:**
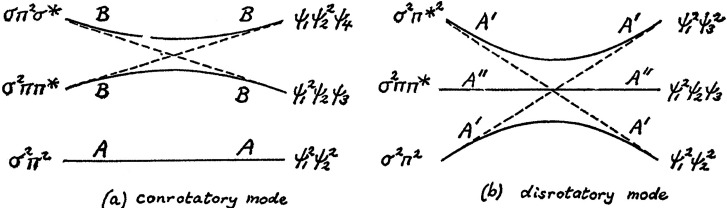
H. C. Longuet-Higgins and E. W. Abrahamson’s state diagram (not orbital correlation diagram) published in March 1965.^57^ The symmetries of the states are listed to the left of each graph) to explain thermally allowed (a, conrotatory) and forbidden (b, disrotatory) cyclobutene ⇋ 1,3-butadiene transformations. Reproduced with permission from ref (57). Copyright 1965 American Chemical Society.

Motivated by private discussions with Woodward in the fall of 1964 in England, the Cambridge University theoretical chemist H. Christopher Longuet-Higgins together with sabbatical visitor E. W. Abrahamson applied correlation diagrams to the cyclobutene ⇋ 1,3-butadiene transformations (Figure 2).^57,58^ The lines here are for state correlations, showing the conrotatory thermal transformations in Figure 2a. The ground state correlates directly (allowed), but the first excited state has a very high energy barrier (forbidden). The dashed lines indicate that orbital crossings occur but are avoided (avoided crossing) at the state level, since two states of the same symmetry mix and avoid crossing. The disrotatory transformations are shown in Figure 2b for the forbidden thermal cyclobutene ⇋ 1,3-butadiene transformations. Here, the orbital crossing of HOMO to LUMO and vice versa would cause the ground state to correlate with a doubly excited product state, but avoided crossing at the state level leads to a significant energy barrier for this forbidden reaction.

**Figure 3:**
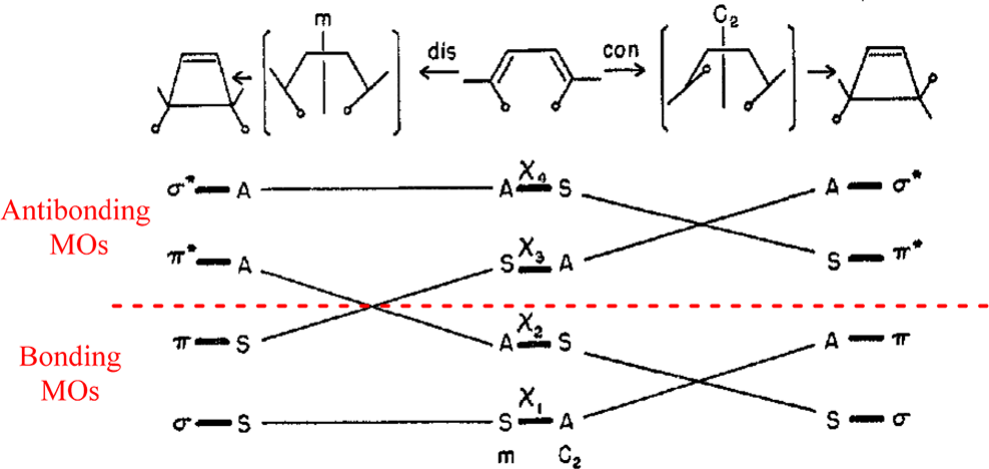
Correlation diagram of the disrotatory (left) and conrotatory (right) 1,3-butadiene ⇋ cyclobutene transformation. This graphic was taken from Woodward and Hoffmann’s 1968 review in *Accounts of Chemical Research* which was entitled *The Conservation of Orbital Symmetry*.^8^ The text and dashed line in red were added. For a thermal reaction, the HOMO and HOMO–1 remain within the bonding region for a conrotatory mode. For the disrotatory mode, the HOMO of 1,3-butadiene correlates with an antibonding MO of the product, indicative of a high activation barrier. Note: Figure 2 shows the corresponding state diagrams for these reactions, with conrotatory motion on the left and disrotatory motion on the right. Reproduced with permission from ref (8). Copyright 1968 American Chemical Society.

Correlation diagrams, first used in the late 1920s^59^ and early 1930s,^60−62^ relate the molecular orbital energy levels of molecules undergoing conformational changes (e.g., as in a Walsh diagram^24,25,63^) or chemical reactions (e.g., as in valence isomerizations^64^). In early February 1965, Hoffmann began using correlation diagrams to distinguish between allowed and forbidden pathways in electrocyclizations. For reactions, the orbital energies are often categorized by certain symmetry elements, e.g., a mirror plane for the disrotatory motion in the 1,3-butadiene ⇋ cyclobutene transformation (Eq 1d, Figure 3).

In the following sections, we present the results of quantum mechanical calculations on a number of allowed and forbidden reactions, including several pericyclic W–H-forbidden reactions that are experimentally observed. These include IRCs that we (the Houk group) have computed for reactions reported by Joseph Michl and J. Kolc in 1971^20,65^ and Horst Prinzbach et al. in 1978.^66^ In doing so, we continue the Longuet-Higgins/Woodward–Hoffmann tradition of using correlation diagrams, IRCs, and related quantum chemical methods to examine allowed and forbidden pericyclic reactions. Indeed, our major focus will be following the orbitals during what would be considered to be W–H forbidden reactions.

## 4 Computed Correlation Diagrams and the Woodward–Hoffmann Selection Rules

In this section, we report the exploration of allowed and forbidden electrocyclizations using modern quantum mechanical methods. In this study, we located the transition state for each reaction and then calculated Fukui’s IRC in order to connect reactant and product. We set the stage by examining first a simple allowed reaction, and following that, by looking at a forbidden one.

### 4.1 Conrotatory (W–H Thermally Allowed) Cyclobutene ⇋ 1,3-Butadiene (Eq 1c) and Disrotatory (W–H Thermally Forbidden) Cyclobutene ⇋ 1,3-Butadiene (Eq 1d)

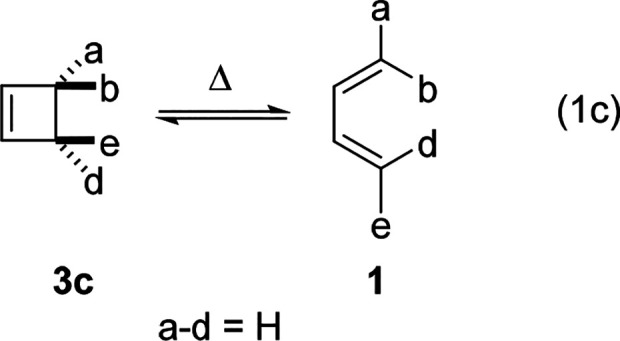



**Figure 4:**
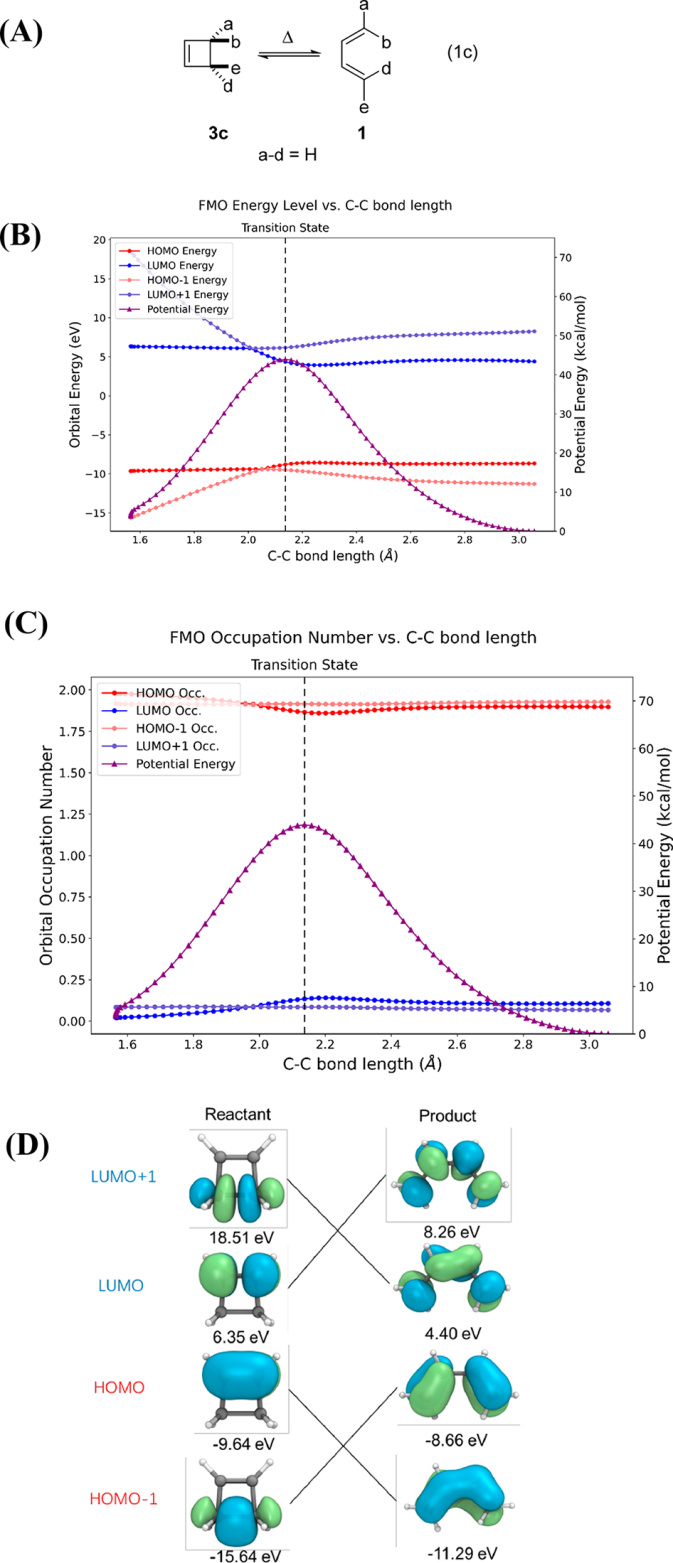
(A) The allowed conrotatory four-electron electrocyclization of cyclobutene ⇋ 1,3-butadiene. (B) IRC with relative potential energy along the intrinsic reaction coordinate, shown in purple. Note the different energy scales in the two ordinates. (C) Orbital occupations of HOMO and LUMO along the IRC for the thermally allowed conrotatory cyclobutene ⇋ 1,3-butadiene electrocyclization. HOMO and LUMO are shown in red and blue, respectively, and the energy along the IRC is shown in purple. (D) FMOs of the reactant and product along with their symmetry correlations. The MOs for 1,3-butadiene are symmetrical but appear asymmetrical, because butadiene is not planar due to steric repulsion of the 1,4-inner Hs. The product has *C*_2_ symmetry. The energies are calculated by CASSCF(4,4)/def2-SVP.

Figure 4 shows the IRC for the W–H thermally allowed conrotatory cyclobutene ⇋ 1,3-butadiene transformation (Eq 1c) using CASSCF(4,4) calculations, a method in which configuration interaction is included between the highest two occupied and lowest two vacant orbitals, so that these FMOs may be occupied by a fractional number of electrons. (“CAS” refers to the “complete active space” of orbitals and electrons that may change places and have fractional occupations. “(4,4)” indicates that the complete active space is composed of the four frontier orbitals and the four electrons within them. “SCF” refers to self-consistent field. The four orbitals referred to in Figure 4 are Fischer–Coulson orbitals^67^ which are similar to Hückel orbitals. In the CASSCF calculation, there is configuration interaction (CI) involving the orbitals in the active space; these orbitals may have partial occupation of electrons instead of either two or zero electrons, as would be the case in a restricted Hartree–Fock calculation. CI captures the fact that the full electronic wave function is not composed of just one electronic configuration but of a weighted linear combination of different configurations. One of these configurations is the ground electronic state, and the others are different electronically excited configurations.

The W–H allowed concerted reaction pathway involves a smooth correlation of occupied orbitals of reactant to occupied orbitals of product (Figure 4B). As the reaction begins, the C(3)–C(4) σ-bond orbital of cyclobutene rises in energy and becomes the HOMO of the product. The overall correlation of the orbitals of the same symmetry is maintained. The molecular orbital level crossing of occupied levels shown in Figure 4B is identical with that predicted Woodward and Hoffmann’s 1969 qualitative correlation diagram.^9^

Figure 4C shows that the orbital occupations (number of electrons) of HOMO and LUMO along the IRC remain nearly 2 and 0, respectively, which is the same as one expects for a closed-shell calculation. This is reflective of the fact that there is little configuration interaction in such a W–H allowed reaction. In the vicinity of the TS, there is a very slight transfer of electron density from the HOMO to the LUMO, resulting from an admixture of a configuration involving occupation of what is the LUMO in the reactant. Figure 4D is the correlation diagram showing nature of the FMOs with the blue-green scheme adopted in 1969 by Woodward and Hoffmann to represent + and – phases of atomic orbitals in each MO.

Figure 4 provides the opportunity to point out that the orbital correlation diagrams of allowed reactions do not reveal the origin of the significant potential barriers that actually can occur during the reactions. Raymond Firestone suggested that this meant that concerted W–H allowed reactions should have very low barriers, or none at all!^68^ However, Figures 4B and 4C show the computed energy barrier of the conrotatory ring opening of 38.5 kcal/mol, which is similar to 35 kcal/mol determined experimentally. Indeed, many allowed hydrocarbon pericyclic reactions have barriers of this magnitude.^69^ In contrast, the Longuet-Higgins and Abrahamson state correlation diagram in Figure 2 shows the energy staying approximately constant from the reactant to product. This is because the symmetry-derived orbital correlation and state correlation diagrams do not include energy changes that occur during a reaction, such as those produced by stretching bonds or increased or decreased strains that involve other orbitals than the orbitals involved in the correlation diagrams.

Distortions of a reactant are required to achieve the transition state geometry, whether or not a reaction is allowed or forbidden, and forbiddenness adds a further energetic penalty to achieve the transition state. An example of this is Rondan and Houk’s use of the behavior of FMOs on the cyclobutene conrotatory electrocyclic ring opening to develop an understanding of factors controlling “torquoselectivity,” the preference for one of the two possible electrocyclic reaction pathways of substituted cyclobutenes.^70^ They showed that two stereoisomeric allowed reactions could have very different barriers due to secondary orbital interactions. Primary orbital interactions only explain why reactions choose an allowed path; they do not discriminate between multiple allowed pathways. The detailed consideration of distortion energies of reactions and their influence on barriers is the subject of the Distortion/Interaction–Activation Strain Model developed by Houk and Bickelhaupt independently, but presented now as the united D/I-AS model.^71^

Everything we have seen in the just discussed W−H allowed model case was as expected. We now turn to a reaction constrained to proceed in a forbidden way, in the case at point, the forbidden disrotatory ring opening of cyclobutene. This reaction was previously studied by Sakai, who found a second-order saddle point (SOSP) for the W–H forbidden disrotatory ring opening TS of cyclobutene (Eq 1d). Here, one negative force constant leads to 1,3-butadiene and the other to a diradical, allyl-methylene.^72^ Sakai also studied a number of allowed and forbidden pericyclic reactions with his CASSCF method.^73,74^
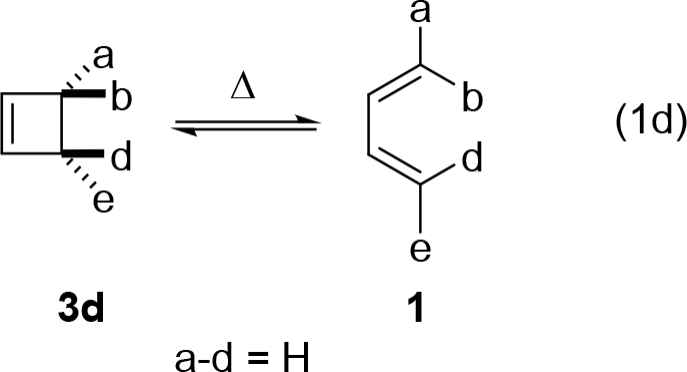


**Figure 5:**
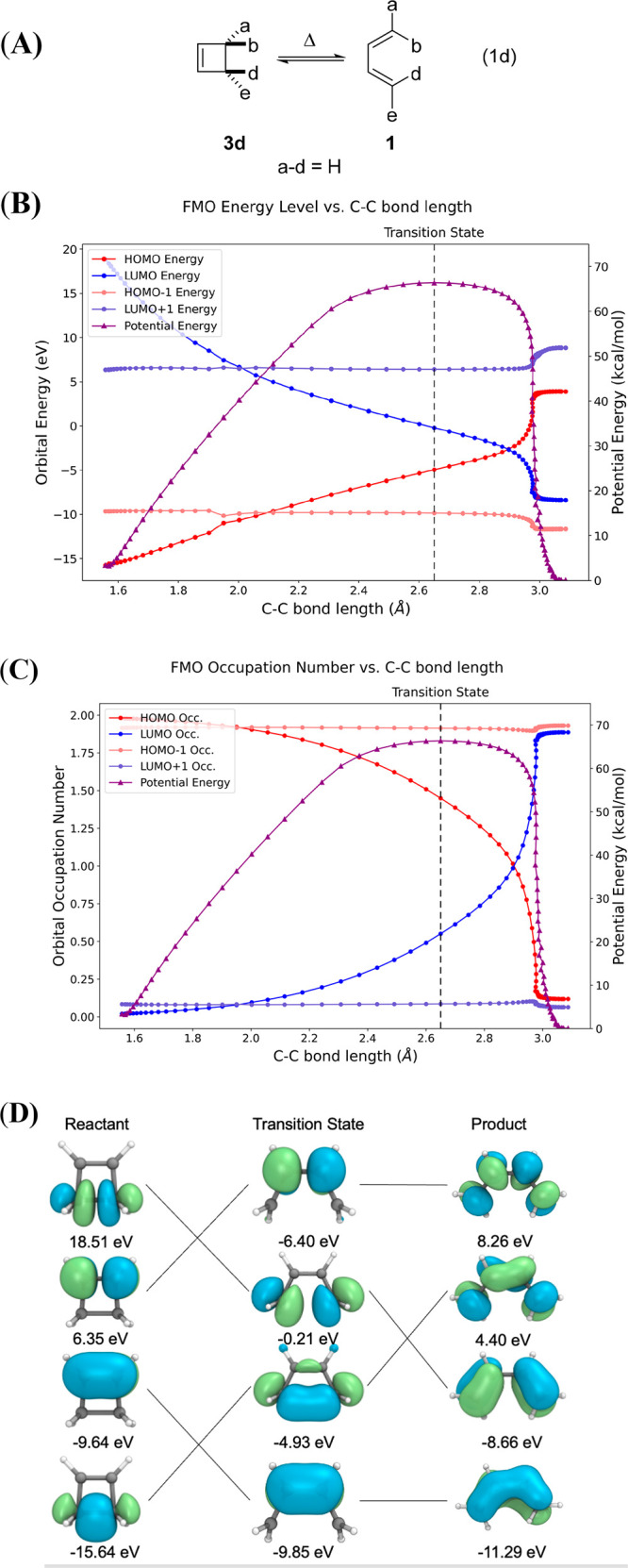
(A) The forbidden disrotatory four-electron electrocyclization of cyclobutene ⇋ 1,3-butadiene. (B) Potential energy (purple) and four frontier orbital energies along the disrotatory cyclobutene ⇋ 1,3-butadiene pathway, maintaining a bisecting plane of symmetry and passing through a second-order saddle point. (C) FMO occupation calculated by CASSCF(4,4)/def2-SVP along the C_s_ IRC. (D) Frontier orbital shapes at reactant, transition state, and product geometries calculated by CASSCF(4,4)/def2-SVP.

Figure 5B shows the energetics of the orbitals along the IRC from cyclobutene to the disrotatory SOSP, and then to butadiene; Figure 5C shows the occupation of the various frontier orbitals along this surface, and Figure 5D shows the orbital correlations and frontier orbital plots. Note in Figure 5B, the broad flat region of the potential energy surface located before, during, and after the SOSP, a region with significant diradical character of the wave function, as reflected in the narrow HOMO–LUMO gap in Figure 5B, and partial occupation of the HOMO (1.75 to 1.00 electrons) and LUMO (0.25–1.00 electrons) in this flat region (Figures 5B and 5C) as the electronic structure reorganizes and electrons are gradually promoted into an orbital conducive to bonding in the product. Also note the separation of the position of the TS (from the top of the IRC) and the HOMO–LUMO crossing point, since the maximum potential energy depends not only on the HOMO energy but also on the energies of all the other occupied orbitals and conformational strain.

These CASCCF(4,4) calculations provide a simple computational way to explore transition states for electrocyclic reactions involving open-shell character and are relatively easy to understand as well. To provide a more nearly state-of-the art treatment of these two reactions, we also performed CASSCF calculations with a larger active space (12e, 12o) and more complete basis set (def2-TZVP). The results of these computations are almost identical to these CASSCF(4,4) calculations (Table 1). Larger active space and basis sets did not lead to any meaningful deviation of the occupation number. The largest difference is in the LUMO of the TS of the disrotatory reaction, where CAS (4,4)/def2-SVP overestimated the occupation number by 0.06. The orbital energies are also in general consistent, while larger deviations were found for the FMOs of reactants and product (cyclobutene and 1,3-butadiene), but the overall conclusion remains the same. Therefore, we believe that CAS (4,4)/def2-SVP is accurate enough to give both qualitatively and quantitatively correct results for our studies. For the reaction energy, our results are also acceptable compared to high accuracy computation and experimental values,^75−78^ where our result is around 5–6 kcal/mol higher than the reference values.

**Table 1:** Occupation Numbers and Energies for Conrotatory and Disrotatory Ring Opening and Conrotatory Reaction Energy Barriers^a^

		HOMO–1	HOMO	LUMO	LUMO+1
**(A) Conrotatory cyclobutene ring opening**					
**Electron occupancy**					
Reactant		1.98	1.92	0.08	0.02
Reactant (12,12)		1.97	1.92	0.08	0.02
TS		1.92	1.86	0.13	0.09
TS (12,12)		1.92	1.88	0.12	0.08
Product		1.93	1.90	0.11	0.07
Product (12,12)		1.93	1.91	0.10	0.06
**Energy (eV)**					
Reactant		–15.64	–9.64	6.35	18.52
Reactant (12,12)		–13.97	–9.53	6.57	18.13
TS		–9.49	–8.80	4.35	6.18
TS (12,12)		–9.40	–8.83	4.78	6.38
Product		–11.30	–8.67	4.41	8.26
Product (12,12)		–11.29	–8.66	4.86	8.76
**(B) Disrotatory cyclobutene ring opening**					
**Electron occupancy**					
Reactant		1.97	1.92	0.08	0.02
Reactant (12,12)		1.97	1.92	0.08	0.02
TS		1.92	1.45	0.55	0.09
TS (12,12)		1.92	1.50	0.49	0.08
CP		1.90	1.01	0.99	0.09
CP (12,12)		1.91	1.02	0.98	0.09
Product		1.93	1.89	0.12	0.06
Product (12,12)		1.94	1.90	0.10	0.06
**Energy (eV)**					
Reactant		–15.73	–9.64	6.35	18.83
Reactant (12,12)		–14.10	–9.53	6.45	18.18
TS		–9.85	–4.94	–0.21	6.40
TS (12,12)		–9.85	–4.93	–0.21	6.40
CP		–10.10	–2.64	–2.68	6.46
CP (12,12)		–10.04	–2.64	–2.66	6.62
Product		–11.65	–8.39	3.91	8.85
Product (12,12)		–11.66	–8.42	4.58	9.51
**(C) Energy barriers of the conrotatory cyclobutene ring opening**					
**Method**	**wB97X-D/6-31G(d)**	**Wn-F12^78^**	**W1^77^**	**CBS-Q837**	**Experimental^75−77^**
Δ*E*^‡^	40.2	35.3	35.3	33.7	33.6 ± 0.2

a (A) Occupation numbers and energies of natural orbitals calculated by CASSCF(4,4)/def2-SVP and CASSCF(12,12)/def2-TZVP for the conrotatory cyclobutene ring opening reactant, TS, and product. (B) Occupation numbers and energies for the disrotatory cyclobutene ring opening reactant, TS, crossing point (CP), and product. (C) Energy barriers of the conrotatory cyclobutene ⇋ 1,3-butadiene ring opening reaction calculated by high-level composite methods and measured experimentally. All values are zero-point exclusive and are in kcal/mol

### 4.2 What is Going on in the Forbidden Reaction?

The level crossing creates a typical diradical situation: two levels close to each other in energy, and two electrons to place in them. This bonding situation has been analyzed many times in the literature, perhaps most pertinently by Salem and Rowland,^79^ Borden,^80^ and by T. Stuyver et al.^81^ This open-shell singlet state is one of four states for a diradical, and the others are a closed-shell singlet and also an open-shell singlet and a triplet.

The ground-state surface on which an allowed pericyclic reaction is moving in this study is a closed-shell singlet that is mainly described by a configuration with two electrons in the lower energy orbital, with an admixture (growing as one passes through the energy maximum) of a configuration with two electrons in the higher level. As a reviewer remarked, the ground state configuration is not inherently polar but is highly polarizable. This was an important early insight of Salem and Rowland.^79^ Building in an electronic enhancement of that polarity, we will see in time as one of the ways of facilitating an otherwise “forbidden” stereochemical reaction.

We turn to the allowed six-electron electrocyclization next, in somewhat less detail.

### 4.3 Disrotatory (W–H Thermally Allowed) *Z*-1,3,5-Hexatriene ⇋ 1,3-Cyclohexadiene (Eq 2d)

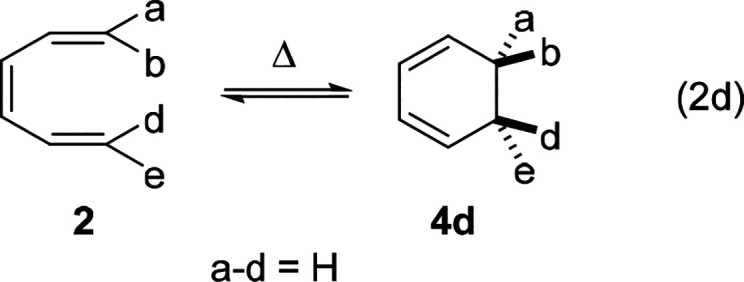



**Figure 6:**
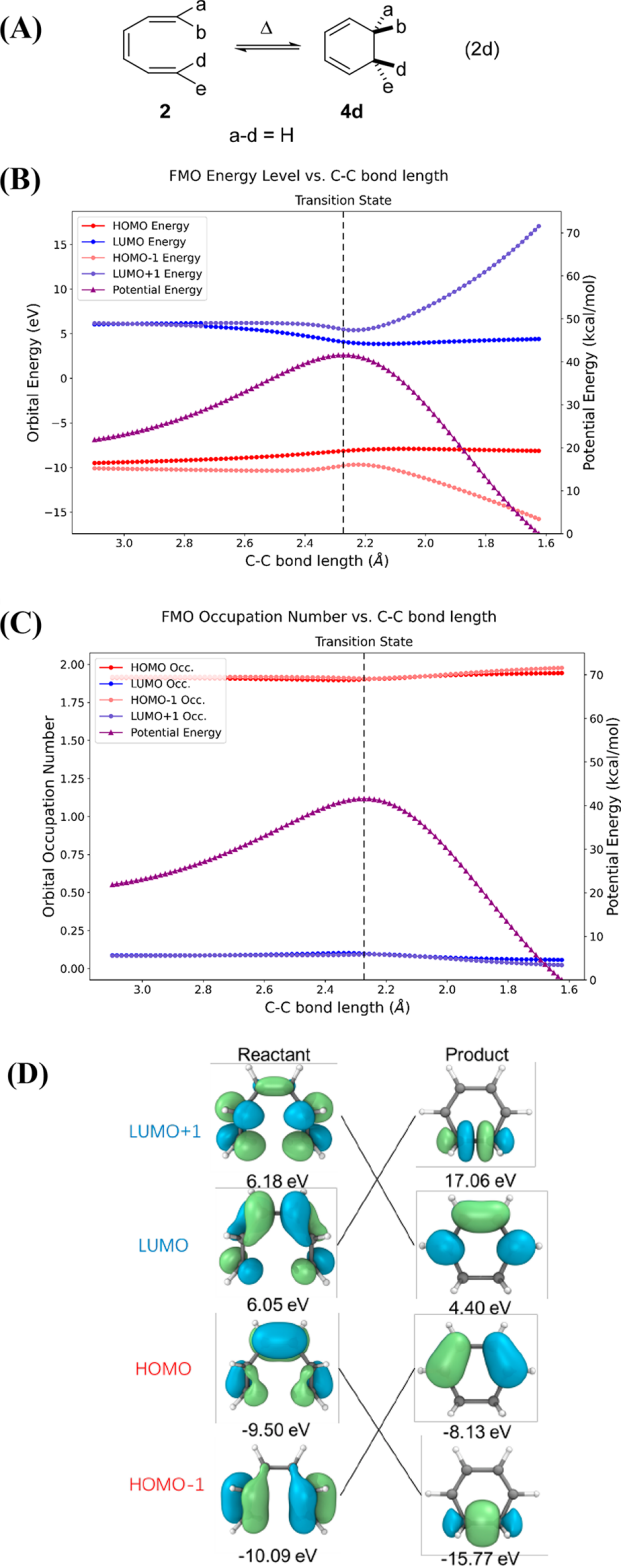
(A) An allowed disrotatory six-electron electrocyclization of *Z*-1,3,5-hexatriene ⇋ 1,3-cyclohexadiene (Eq 2d). (B) Correlation diagram including the molecular orbital energies along the allowed disrotatory electrocyclic ring closing of *Z*-1,3,5-hexatriene ⇋ 1,3-cyclohexadiene and energy of reaction. (C) Orbital occupation of FMOs along this reaction path and the energy of the reaction. Calculated by CASSCF(4,4)/def2-SVP. (D) FMOs of reactant and product calculated at the CASSCF(4,4)/def2-SVP level of theory.

The thermally allowed disrotatory electrocyclizations of *Z*-1,3,5-hexatriene ⇋ cyclohexadiene (Eq 2d) were next examined using CAS(4,4)/def2-SVP calculations (Figure 6). The HOMO and LUMO of this transformation are slightly closer in energy in the vicinity of the TS, but they do not cross. In the CASSCF result shown in Figure 6C, the LUMO remains almost entirely unoccupied for the duration of the reaction, and the HOMO remains doubly occupied. There is some slight configuration interaction from the HOMO to the LUMO, mostly in the vicinity of the TS. The occupied MOs of *Z*-1,3,5-hexatriene smoothly correlate with the occupied MOs of 1,3-cyclohexadiene: The LUMO of the reactant correlates with the LUMO+1 of the product, and the LUMO+1 of reactant correlates accordingly with the LUMO of product. The reactant’s HOMO correlates with the product HOMO–1, and HOMO–1 correlates with HOMO, but their crossing is located out of the scale in Figure 6B; the plotted region in Figure 6B begins after the crossing. The patterns in Figure 5 follow what were proposed in the early publications on orbital symmetry control^8,9,57^ and in numerous textbooks^82^ based on fundamental principles and eHT calculations.

Our previous consideration of a forbidden (disrotatory in that case) four-electron electrocyclization, informs us as to what we can expect for a forbidden (now conrotatory) six-electron process.

### 4.4 Conrotatory (W–H Thermally Forbidden) 1,3-Cyclohexadiene ⇋ *Z*-1,3,5-Hexatriene (Eq 2c)

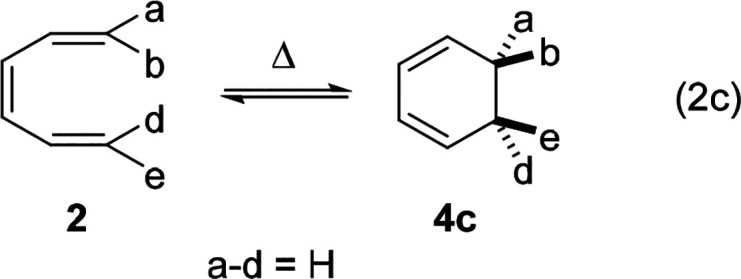



**Figure 7:**
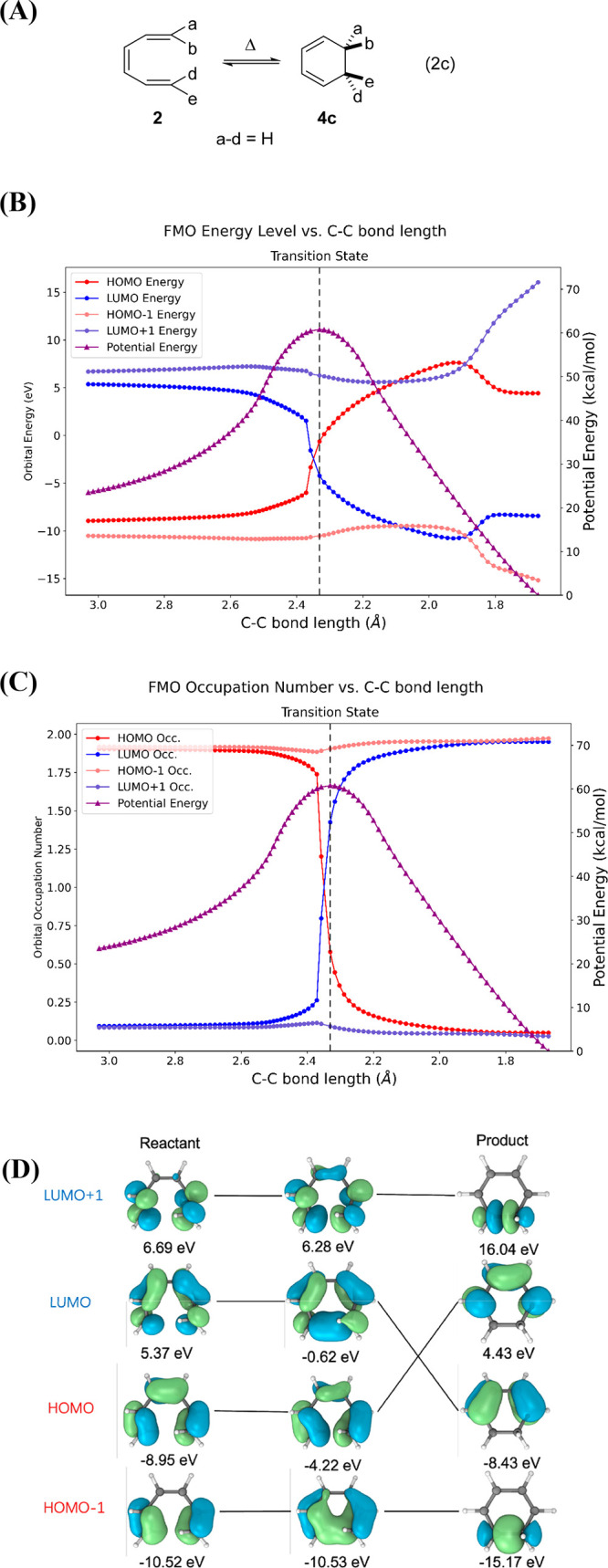
(A) Thermally forbidden conrotatory six-electron electrocyclization, Eq 2c. (B) Correlation diagram including the molecular orbital energies. (C) Orbital occupation of FMOs along this reaction path, calculated by CASSCF(4,4)/def2-SVP. (D) FMOs of reactant, crossing point, and product calculated by CASSCF(4,4)/def2-SVP.

Computational results for the thermally W–H forbidden conrotatory electrocyclization of 1,3-cyclohexadiene ⇋ *Z*-1,3,5-hexatriene (Eq 2c) are provided in Figure 7. This reaction was studied earlier by Sakai.^72−74^ We enforced *C*_2_ symmetry along a conrotatory reaction pathway, whereas the lowest energy conrotatory pathway would go through a less symmetrical transition state.

A high-energy transition state with substantial diradical character is formed in Eq 2c, with single occupation of the degenerate orbitals. The IRC reveals an absence of an intermediate in this transformation, i.e., this is an enforced W–H thermally forbidden reaction that computationally is shown to be concerted (Figure 7B). Similar results were previously reported by Sakai^72−74^ and by Sekikawa et al.^83,84^

The orbital occupation values from this CASSCF calculation are shown in Figure 7C. The orbital occupation values change gradually until they reach the region of the TS when the electronic wave function changes from reactant-like to product-like. The electrons switch from the HOMO to the LUMO as the reaction passes through the TS (Figure 7D). Here the CAS wave function has one electron in each orbital; this is an open-shell singlet diradical.^81^ Within the formalism of the correlation diagram for the conrotatory (W–H thermally forbidden) 1,3-cyclohexadiene ⇋ *Z*-1,3,5-hexatriene system, the original HOMO of the starting compound becomes the LUMO of the product, and the original LUMO of the starting compound becomes the HOMO of the product. This forbidden reaction occurs in a concerted fashion^62^ via a high energy barrier 15 kcal/mol above that on the allowed disrotatory path.

We have set the stage with a blow-by-blow discussion of prototype allowed and forbidden electrocyclic reaction pathways, analyzed with contemporary CI-SCF methodology. Now let's look at what has been learned of the various ways in which forbidden reaction pathways can be induced to become the experimentally preferred one.

### 4.5 Prinzbach’s Vinylogous Sesquifulvalene (5): A Woodward–Hoffmann Forbidden but Favorable Reaction (Eq 3c)

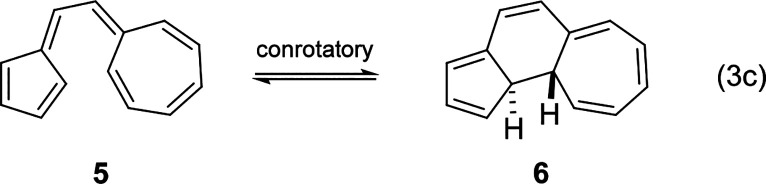



**Scheme 1:**
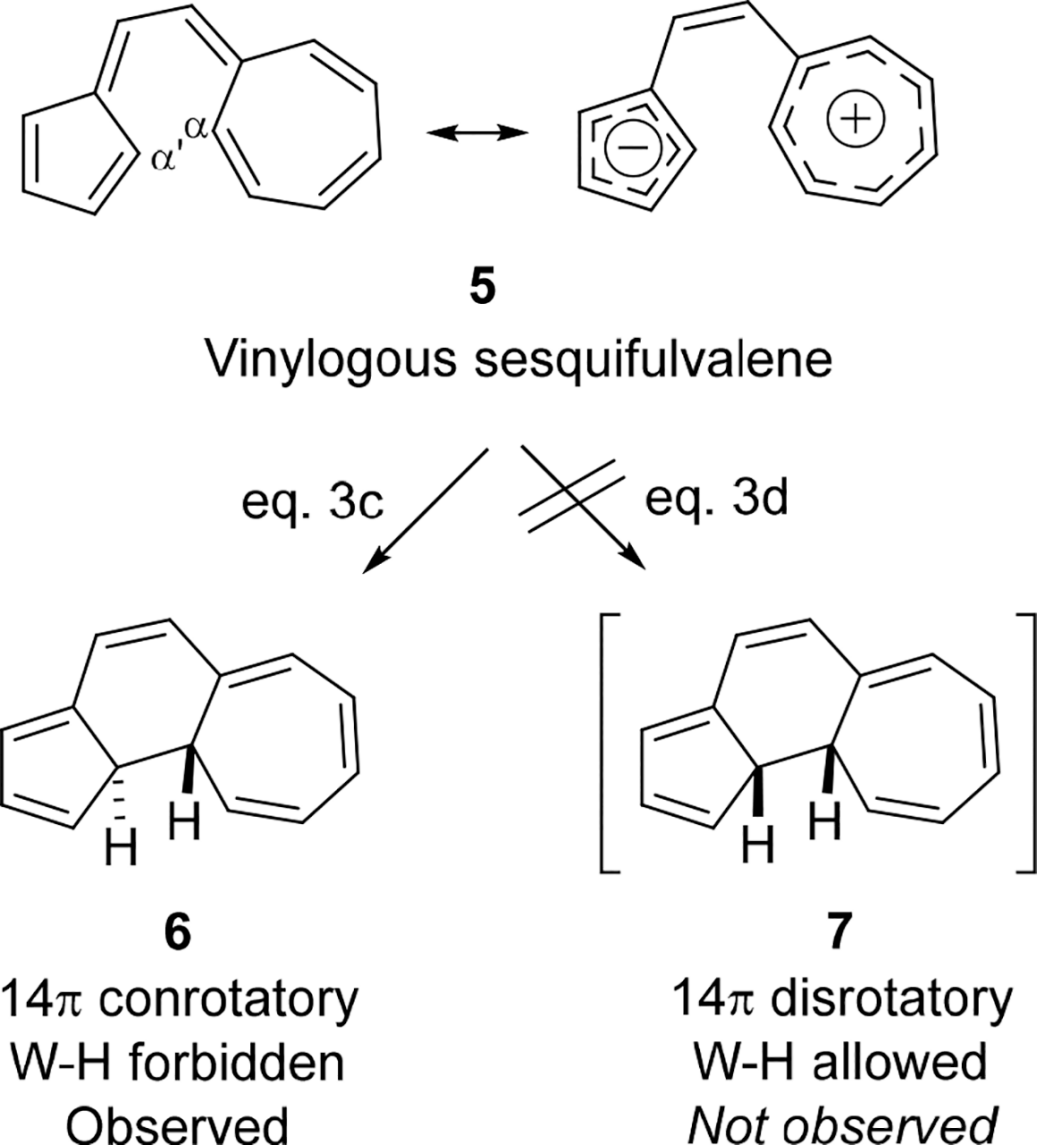
Thermally Forbidden Electrocyclization of the Vinylogous Sesquifulvalene **5** to **6** (Eq 3c) Reported in 1978 by Prinzbach et al.^13,66^ Adapted with permission from reference (13). Copyright 2021 American Chemical Society.

Recently, the Houk group explained the electrocyclization of the vinylogous sesquifulvalene **5** (Scheme 1) reported in 1978 by Horst Prinzbach^66^ as a “flagrant violation of the Woodward–Hoffmann rules!”^13^ The transformation **5** ⇋ **6** was found to be an energetically favorable concerted, but W–H forbidden electrocyclization. The Houk group observed a high degree of polarization of the 14-π-electron system **5** which undergoes a W–H forbidden conrotatory cycloaddition resembling the attack of a cyclopentadienide anion onto a tropylium cation to **6** (Eq 3c).^13^ Our calculations showed some degree of charge transfer in **5**, as expected since the cyclopentadienide and tropylium are both aromatic six-electron systems.

**Figure 8:**
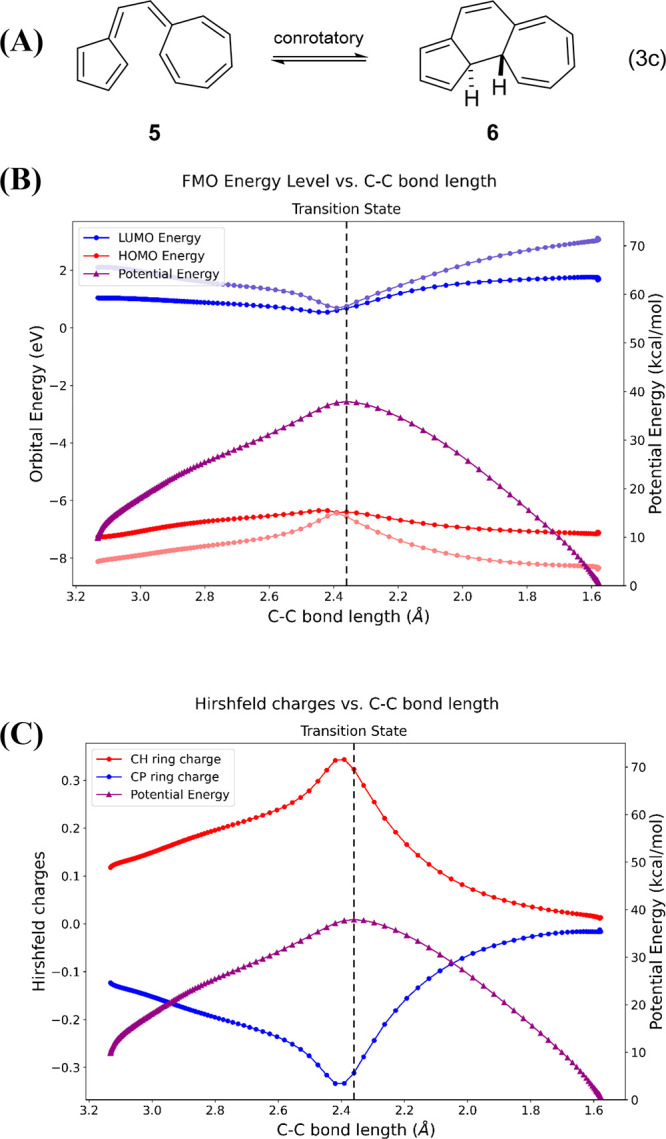
(A) Prinzbach’s W–H forbidden but experimentally favored 14-electron conrotatory electrocyclization (Eq 3c).^13,66^ (B) FMO energies for forbidden but favored conrotatory electrocyclization. (C) Hirschfeld charges for conrotatory electrocyclization. These closed-shell reactions were computed using the Hartree–Fock (HF)/def2-SVP wave function based on ωB97X-D/6-31G(d) optimized geometries.

Figure 8B shows the computed energy diagram for the forbidden but observed conrotatory pathway of the vinylogous sesquifulvalene **5**.^66^ In both electrocyclizations, concerted **5** ⇋ **6** and **5** ⇋ **7**, the polarization of the system increases as the transition state is approached. This zwitterionic character stabilizes the HOMO and destabilizes the LUMO, maintaining their energy separation. Indeed, this internal charge transfer occurs in both the allowed (unobserved) and forbidden (observed) reaction pathways. This conclusion for **5** ⇋ **6** is supported by the Hirshfeld charges^85^ shown in Figure 8C. The Houk group previously described how the experimental observation of the W–H forbidden pathway is due to geometrical preference for the conrotatory chairlike transition state over the disrotatory boat-like conformation.^13^

In these donor–acceptor substituted push–pull systems, the donors and acceptors influence the coefficients in the FMOs. During the reaction, the HOMO becomes localized at the acceptor-substituted terminus, and a node is formed on the donor-substituted terminus. In the LUMO, a node is formed on the acceptor-substituted terminus, and the LUMO becomes localized on the donor-substituted terminus. The node in the HOMO on one of the bond-forming carbons allows the two bond-forming carbons to rotate in either direction with respect to each other. Because there are no longer phased lobes on the terminus with the node, it does not matter which way the carbons rotate. There is effectively a lone pair on the acceptor-substituted terminus, attacking a carbocation at the donor-substituted terminus.

The Prinzbach case is an isolated example, but it is also emblematic of a general strategy for lowering the barrier for forbidden reactions: induce (favor) polarity. Judicious substitution to electron donating and withdrawing substituents at the bond-breaking site is likely to reduce, sometimes substantially, the barrier to bond cleavage in the “forbidden way.” In a separate study, for a substituted bicyclo [2.2.0] hexene, the Houk group will analyze in detail such a process.

### 4.6 Michl’s Pleiadene: A Thermal Disrotatory Woodward–Hoffmann Forbidden Cyclization (Eq 4d)^20,65^

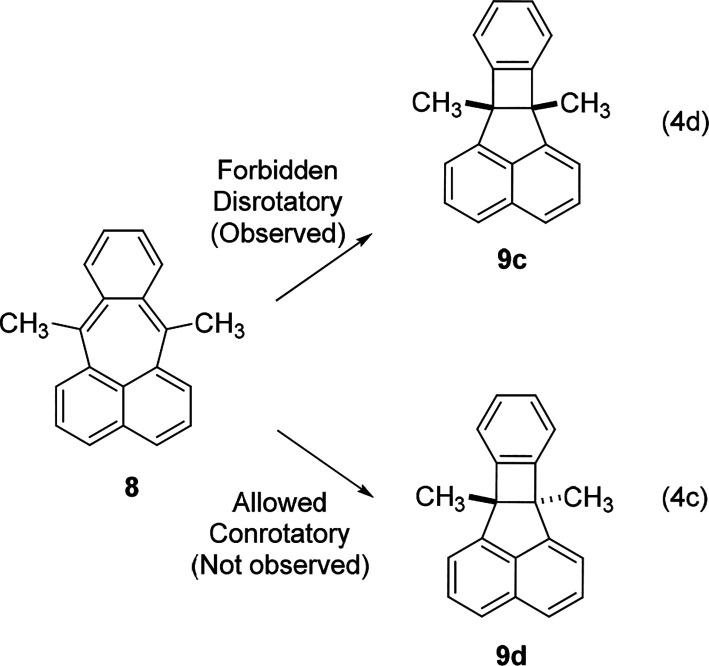



**Figure 9:**
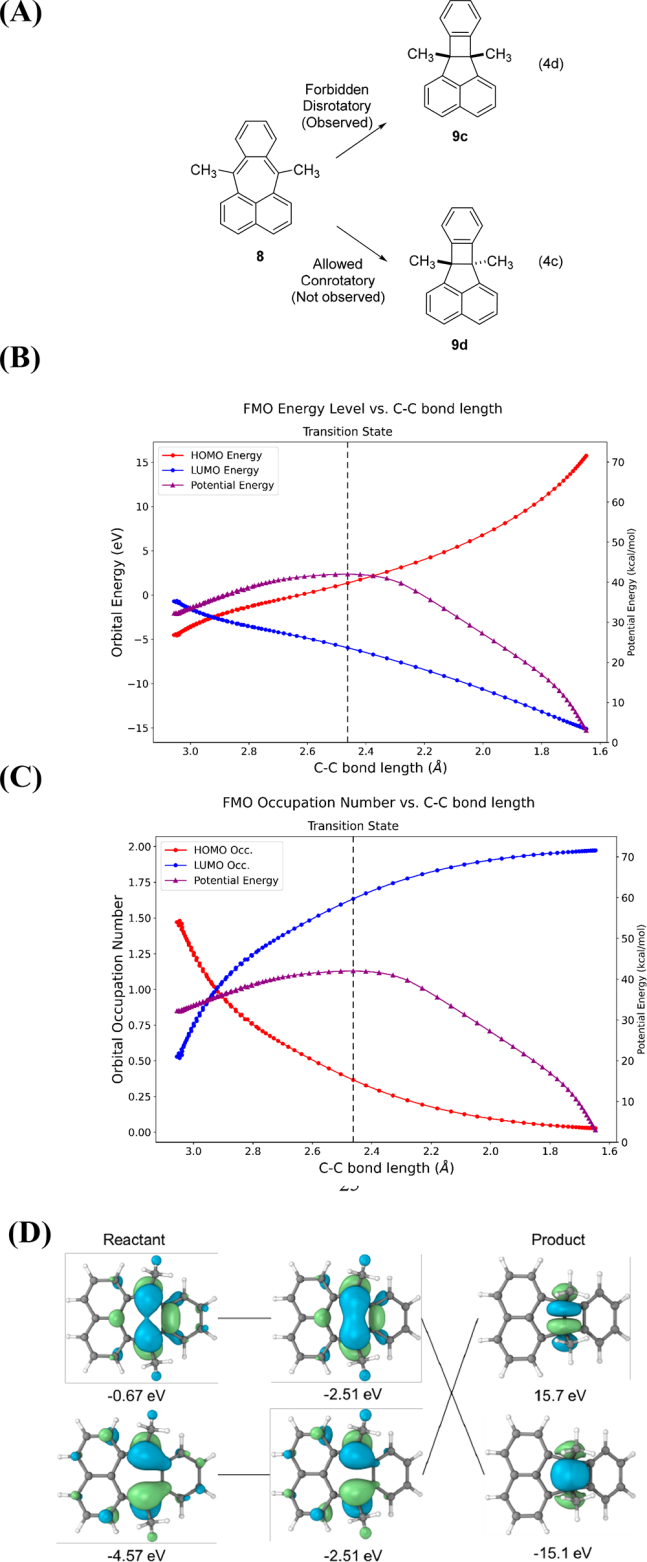
(A) W–H forbidden yet observed electrocyclization of 7,12-dimethylpleiadene (**8**) ⇋ **9**. (B) CASSCF(2,2) potential and FMO energies along the reaction path. (C) MO occupations. In both diagrams, the purple line is the potential energy. (D) HOMO and LUMO visualizations calculated by CASSCF(2,2)/def2-SVP.

Another example of a W–H forbidden cyclization that can occur when forced to by geometric constraints is the 7,12-dimethylpleiadene transformation **8** ⇋ **9c** (Eq 4d in Figure 9A). This disrotatory forbidden cyclization was originally studied experimentally and theoretically by Josef Michl.^20,65^ Our calculations indicate that the observed product **9c** is 52.9 kcal/mol more stable than the product of the allowed conrotatory electrocyclization. This enormous thermodynamic driving force causes the forbidden disrotatory process to occur in contrast to the W–H allowed reaction which has a large energy barrier and its high energy product. The diradical character of the starting material raises its energy as the diradical mixes with the ground state, thereby lowering the relative barrier to the forbidden process.

As can be seen in Figure 9, the forbidden electrocyclization of 7,12-dimethylpleiadene involves intended MO level crossings of the HOMO and LUMO. However, Figure 9B demonstrates that the energies of the HOMO and LUMO are nearly the same in the very early stages of the reaction. Figure 9C shows the diradical character of **8**. Figure 9D shows that the CASSCF wave function of the HOMO is occupied by 1.5 electrons, while the LUMO is occupied by 0.5 electrons. This is about 50% diradical character.^81,86^ The diradical crossing point is easily achieved very early along the reaction path. In the starting material, the LUMO already contains 0.5 electron, from which it must increase to one electron at the crossing point. There is greater CI stabilization of this transition state than in the bicyclo[2.2.0]hex-2-ene system. Once again, in this W–H forbidden reaction, the FMOs cross, but the ground state of the starting material **8** correlates smoothly with the ground state of the product **9c** due to the movement of electrons from the reactant HOMO to what was the reactant LUMO along the reaction path. These orbitals are shown in Figure 9D.

The Michl case, while again a special one, is also a signpost for engineering formally forbidden pathways. Organic chemists are so good at building in strain by bridges and substituents. In this case, the aim might be to make the allowed opening as "uncomfortable" as possible for the molecule, leading to high energy, even impossible products. While a formally forbidden stereochemical course might lead to a more stable product. Elsewhere, we study a prototype for this kind of tuning of a reaction path, in the opening of bicyclo[2.2.0]hexene to cyclohexadiene.

## 5 Additional Discussion and Summary

### 5.1 Recapitulation

**Figure 10:**
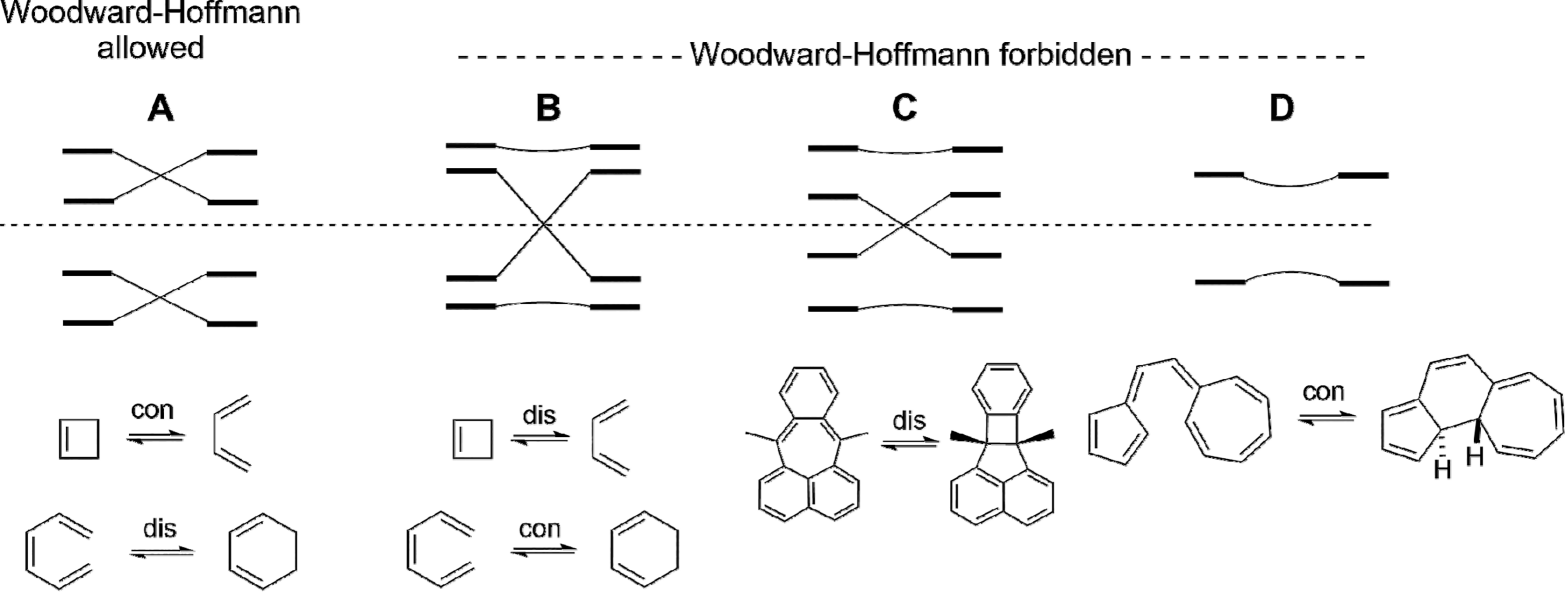
Correlation diagrams for various classes of electrocyclizations shown. The HOMO, HOMO–1, LUMO, and LUMO+1 are shown for each reaction. (A) Allowed reactions according to the W–H selection rules. (B)–(D) Forbidden reactions according to the W–H selection rules. (B) Typical level crossing from bonding HOMO to antibonding LUMO for forbidden reactions according to Woodward and Hoffmann.^9,11^ (C) A highly conjugated system with diradical character that is found to follow a forbidden concerted path, as represented by the Michl case in Figure 9. (D) An avoided HOMO–LUMO level crossing for a W–H forbidden reaction caused by strong polarization induced by donors and acceptors.

A summary of the results of this paper and the types of correlation diagrams for a wide variety of electrocyclizations are shown in Figure 10.

Normal W–H allowed reactions have smooth correlations of occupied orbitals of reactant with those of product within the bonding region, as illustrated in Figure 10A. For thermal electrocyclizations, these are conrotatory for 4*n* electron systems and disrotatory for 4*n* + 2, and the converse is true for photochemical reactions. W–H forbidden reactions have hardly been studied previously, because they do not generally occur experimentally unless induced by geometrical constraints or by photochemical excitation to excited states, which reverses the W–H selection rules.

A typical forbidden reaction correlation diagram in Figure 10B shows the correlation of a reactant HOMO with product LUMO of the same symmetry and vice versa. These transition states are diradicals that have a higher energy, but the reaction can still occur in a concerted (pericyclic) fashion. The lowest energy reaction pathway for a W–H forbidden reaction usually will not follow the disfavored path, because other, lower energy pathways and transition states exist. Figure 10C shows an example of Michl’s 7,12-dimethylpleiadene (**8**, Figure 9) where CI facilitates a reaction forced to follow a forbidden route by geometric constraints. Figure 10D shows a correlation diagram for a reaction in which the high polarity of the molecule’s π-system induces a node at a reacting terminus, causing no orbital symmetry penalty for a formally forbidden reaction. In such a case, there is no orbital symmetry control of reaction stereochemistry, and the formally forbidden pathway can be favored for steric and torsional reasons.

Our perspective concentrates on electrocyclizations, which differ from other pericyclic reactions in that no real diradical or zwitterionic intermediate mechanism has been computationally found. They may not even be possible. Cycloadditions, sigmatropic shifts, and group transfers do have clearly defined stepwise mechanisms with detectable (in principle) intermediates, and these happen always with forbidden reactions but also with allowed reactions that have excellent radical or ion stabilizing substituents.

### 5.2 What This Paper Does

The Woodward–Hoffmann papers, as historical investigations have demonstrated, understood well that correlation diagrams were just a stepping-stone, only one of several, to analyze the continuity of bonding in occupied levels that was really behind a reaction being “allowed”. Woodward and Hoffmann really paid little attention to forbidden reactions: the crossing between filled bonding levels and unfilled antibonding ones was the obvious source of a forbidden barrier. Photochemistry via electronic excitation could overcome this.

Woodward and Hoffmann did not enter the wonderfully complicated field of the ordering and reactivity of the excited states of molecules. They were probably well advised not to enter into photochemical detail. While Luitzen Oosterhoff did so in the late 1960s,^87−89^ real understanding came only later in the revelations of conical intersections of excited and ground state PES, and in an understanding of the complexity of excited and reactive excited states.^28,30,84,90−94^ The complexity in photochemical reactions is manyfold; the state to which a molecule is excited may not be the reacting one, with intersystem crossing and radiationless transitions along the way, for example.

This publication looks at forbidden reactions in substantive detail, an approach not taken by Woodward and Hoffmann and only rarely since, most notably by Sakai.^72−74^ We begin by constructing reaction surfaces for forbidden four- and six-electron electrocyclic reactions and contrasting them with allowed pathways. New features emerge in the forbidden surfaces, among them a mesa in the potential energy surface for these W–H forbidden reactions. The important detailed analysis of forces along the Diels–Alder reaction pathway by Elfi Kraka and the late Dieter Cremer is also of note in this regard.^95−97^ We examined an interesting potential violation, Michl’s 7,12-dimethylpleiadene thermal disrotatory forbidden cyclization.^65^

## 6 Perspectives

### 6.1 The Triumph of Computational Chemistry

Computational chemistry has become a lively, productive subfield of chemistry, and for its young and not-so-young practitioners, an eminently employable profession.^98,99^ We are far beyond extended Hückel explorations;^53,100^ we have reliable computations of potential energy surfaces of organic, inorganic and biochemical reactions, including complex catalytic cycles and reactions involving hundreds of intermediates. Intrinsic Reaction Coordinates,^101^ Kenichi Fukui’s invention from 1970, are routinely calculated for complicated reactions; some of these reactions are being probed by trajectory calculations, a new approach to organic reactivity.

There is an interesting time point in the offing, for computational chemistry is an obvious target for the ingenious applications of so-called “artificial intelligence” to science. Soon (maybe yesterday), given a training set of correctly quantum-mechanically computed organic reactions, one can program machine learning and neural network machinery to compute pretty accurately organic reaction surfaces. The power of quantum computing will only make this easier (How apropos: to use quantum computing to model quantum chemistry!). Hoffmann and Jean-Paul Malrieu have marked this waypoint provocatively as “Quantum Chemistry, † ca. 2020?”^102^ (The character “†” is the “deceased” symbol used as text within the publication, not a footnote symbol.)

### 6.2 Trajectories and Dynamics

In an earlier section, we outlined the state of the art in the decade after the introduction of the Woodward–Hoffmann rules, which led to an early realization that dynamic calculations, the interaction of various degrees of freedom, and trajectories eventually had to be done. For in the end, with the proper tools still to be developed for interpreting such calculations (and putting them into the hands of organic chemists), this theory would bring an understanding of mixed stereochemical outcomes.

We want to highlight here the work of Barry Carpenter. He began by studying a number of reactions that involve diradical intermediates and traced the dynamic behavior of these species that are often moving in broad flat regions on potential surfaces, lacking bonding between centers that become bonded in the products. Such reactions are not pericyclic, often because the potential thermally allowed pericyclic pathways involve considerable geometrical distortions. Houk and Singleton also pioneered in the dynamics of pericyclic reactions, beginning with the Houk group explorations of a variety of Diels–Alder reactions and a thorough exploration of several Cope rearrangements involving pericyclic and also stepwise mechanisms.^44^

Carpenter’s work is more far-reaching than just explaining the unusual stereochemical outcomes that we mention. It has led him to re-examine the Transition State Theory and the standard kinetic models connected to it. We cannot do better than to quote Carpenter’s research summary:

“. . . it has become apparent that reactive intermediates may exhibit effects that are traceable to breakdowns in some of the key approximations in the standard kinetic models. The symptoms of these effects are that product ratios may not reflect the apparent symmetries of the intermediates from which they are formed, the intermediates may have no well-defined lifetimes, and intermediates may even form products in an oscillatory manner.”^103^

### 6.3 So, Can There Be Forbidden Pericyclic Reactions?

By writing “Violations. There are None!”,^9,11^ were Woodward and Hoffmann just provocative? Psychologically perceptive for sure—their categorical locution immediately engaged the organic chemistry community, and continues to do so, even today, more than 50 years later.^13,71,104−109^

Or were Woodward and Hoffmann realistic, voicing a considered scientific conclusion?

Here is what we think in 2023, 54 years after the long Woodward and Hoffmann treatise and after much good theory and experiment and deep thinking.^34^ Maintaining a degree of bonding in all filled orbitals is necessary in an “allowed” reaction, usually a relatively low activation energy process. As one follows the evolution of the MOs in a reaction, the real or intended correlation of a filled reactant level to an unfilled antibonding one is the clearest signal of a high-activation-energy “forbidden” reaction.

As discussed in this publication and elsewhere,^13,105,110^ it is possible to design features in molecules and transition states that preclude or disfavor geometrical achievement of an allowed TS. It is also possible to endow a “forbidden” TS with substantial diradical or ionic character (electronic asymmetry, strong donor/acceptor polarization) at the bond-forming loci.

Where such designed (or naturally occurring) strong electronic asymmetries occur (and this paper collects a number), we think the reactions should be said to be *outside of Woodward–Hoffmann control*, *a way to say that the Woodward–Hoffmann rules do not apply*.

But, if you recognize the strangeness of such unique reactions, you can call them “violations.” We will not get in your way as reviewers. And if you demand that there be a violation in such a case, *we would delineate an enunciation of the two types of violations that may occur regarding the W–H rules. Violations of the first order are of the criteria necessary for a reaction to be governed by the Woodward–Hoffmann selection rules. Violations of the second order are of the selection rules themselves*.

Organic chemists have expended considerable intellectual effort in exploring “forbidden” reactions, both experimentally and theoretically. The examples discovered by Prinzbach^66^ and Michl,^65^ and the hypothetical donor–acceptor substituted cases studied by the Houk group^13,73^ violate the prediction from the W–H rules that electrocyclic reactions involving bonding changes in a cyclic system of 4*n* + 2 must be conrotatory. Violations have been established, and why and how they deviate has been explained. We have also alluded to how the mixed stereochemical cases have led to a renewed valuation of reaction dynamics.

Woodward and Hoffmann were bold to offer actual rules for pericyclic reactions, and we think this is the feature that caused such interest by organic chemists rather than Woodward and Hoffmann’s observation that reactions follow the lowest energy pathway. Nonetheless, the lowest energy path remains the one that maintains some bonding in all filled orbitals. We understand that it is sometimes easy to establish this and sometimes devilishly difficult. The figures in this publication reveal continuous bonding, even in several “forbidden” reactions.

### 6.4 Going Forward: Strategies for Effecting “Forbidden” Reactions

The above section notwithstanding, we now think about the future. From cases previously studied by the Houk group as well as by others, there have emerged a number of instances where formally forbidden reaction paths emerge or can be “encouraged” to become real. Here is the beginning of a descriptive etiology of several such cases.

#### 6.4.1 Dynamic Effects, and Their Manipulation

As we mentioned in the course of this article, early examples of incomplete stereocontrol, or “mixed” stereochemical outcomes, led Carpenter and others to an incisive analysis of dynamic effects. Temperature dependence of the stereochemistry and large entropies of activation in a classical analysis are associated with such effects. Can the understanding we have of dynamic effects at this time be parlayed into design principles of synthetic import? We are not sure; it is clear that pieces of understanding are falling into place. For example, pulsed pumping in specific, quantized, vibrational modes, or in an assembled combination of pulses that approximates a reaction channel, remains a grail of chemical physics. One day, someone will figure out how to do this.

#### 6.4.2 Mechanochemical Prompting of Forbidden Reactions

The molecular engineering needed to apply precisely measured (e.g., in piconewtons or even nanonewtons) mechanical forces to tethered molecules has been realized. One can force a molecule to do a forbidden reaction and measure precisely that coercion. What remains to be done is turn the selective knowledge we have gained into synthetic methodology on a molar scale.

#### 6.4.3 Thermodynamics

Always there, and if the exothermicity of a reaction is large enough, it will have its effects on the activation energy as well. If thermochemical driving is combined with a reduction in volume (most apparent in bimolecular processes, but also in other pericyclic reactions), otherwise forbidden pathways may be activated under compression.^111^

#### 6.4.4 Molecular Constraints

This is both fun and the exercise of the organic chemist’s synthetic bravado. With atomic balls and chains, encumberments of the most exquisite type, and even engineered attractive interactions (ionic, dispersion forces), one can make life very uncomfortable for a reaction path for an allowed reaction and sterically and electronically attractive for a reaction trajectory that follows the forbidden route.

#### 6.4.5 Ionic Byways

The Prinzbach vinylogous pleiadene analysis (Figure 9)^66^ showed clearly how a system, stacked to favor ionicity along a reaction coordinate, took advantage of just that possibility. This is a way to circumvent forbiddenness by a judicious flow of electrons. Could one induce this with newly revived organic electrochemistry?

#### 6.4.6 Stabilizing Alternatives

In a way, those systems in which substitution would favor a diradical (e.g., the Michl systems^20,65,86,112^) provided examples of electronically facilitating an alternative to a forbidden reaction. Much earlier, we saw examples of radical stabilization clearly diverting a pericyclic process to a nonconcerted path–we are thinking here, for instance, of the effect of phenyl substitution on the Cope rearrangement.^113,114^ Unexplored opportunities for using this strategy remain, for instance, using the captodative effect as a substitution pattern.

### 6.5 The Evolution of Computational Exploration of Organic Reaction Mechanisms

We’ve already mentioned the triumph of computational chemistry in our time. It is appropriate here to give credit to John Pople^115−118^ and Paul von R. Schleyer,^119−122^ two very different scientists who in their own way propelled the field to its present significance, not to mention a pantheon of great computational chemists who gave us the quantum mechanical methods we use today.

Computers are 100 billion times faster now than they were in the 1970s. It has gradually become possible to explore chemistry computationally and numerically with good (chemical) accuracy (±1 kcal/mol or even ±1 kJ/mol). The computational power now available makes it possible to explore unknown chemistry and make quantitative predictions. Quantum computing is around the corner. We have mentioned in passing the remarkable capabilities offered by applying machine learning and neural network methods to chemical problems.

Computational power has made it possible for theory to rationalize complex reactions but also predict reliably, and the latter is becoming a more prominent feature of computation. Because of this, the borderline between computation and experiment has shifted. So has the makeup of groups that pharma deploys to bring to the market a new drug. If anything, the universe of computational chemistry is far, far more expansive than the universe of experimental chemistry. This is so for obvious reasons: not only can computational chemistry study real compounds and real experiments, but computational chemistry also^123−125^ can study imaginary compounds and imaginary reactions under imaginary conditions!

Can the cornucopia of computational results, as many and as accurate as they promise to be, create new theoretical models for chemistry? Conceptual density functional theory has done so, giving us new measures of reactivity. But on what we are gaining from the application of artificial intelligence to chemistry, the verdict is still out. Let us see what happens.

Has anything changed with respect to pericyclic reactions today relative to 1969? For simple allowed reactions, along with the advances of computational chemistry, we are able to calculate the activation energies and thermochemistry of such a reaction accurately. For unusual situations, the 7,12-dimethylpleiadiene (**8**) being an example, we can identify the factors that cause low activation energies in formally W–H forbidden reactions. We have learned a lot about bifurcations and branches in reaction paths and their stereochemical consequences.

### 6.6 Where Will We Be? Looking into the Near-term Future

First, for ground state reactions, we believe we have had a basic understanding of what makes a reaction Woodward–Hoffmann allowed or forbidden for some time. And that we have some understanding as to how to modify the barriers for allowed reactions and what we need to do to lower the energy of the transition state for forbidden reactions. But some developments are still to come, both experimental and theoretical.

The transition states of some forbidden reactions have biradical character, as we have demonstrated in this publication. For some time, we have known how to stabilize diradicals and diradicaloids, in particular, by captodative substitution, a strategy well-honed in the design of candidates for singlet fission.^81^

We have demonstrated how developing ionicity in a transition state region can lower the energy of that part of the phase space. We need to learn how to make good use of tailored ionic liquids as solvents or how to vary the ionicity of a solvent over the course of a reaction, so as to make this a way to adjust the energy of the TS for a reaction. To achieve oriented polar effects may require electric fields or attachment to surfaces.^126^

Some of the unusual patterns we have discerned in computationally derived PESs are worth exploring; they may have consequences for the outcomes of reactions. In the forbidden reactions such as Eq 1d (Figure 5), a flat-topped region, a mesa, near the transition state region was found. Might this be related to “twixtyls” discussed by Hoffmann,^127,128^ Michl,^65^ and Herndon^129^ years ago and more recently by Tantillo?^130^

### 6.7 What’s Ahead?

The thrill of learning that pericyclic reactions are catalyzed by enzymes, long-imagined, but only proven in this century, has led to a new class of enzymes, the pericyclases, named by Tang and Houk.^99^ These are now known to catalyze cycloadditions, ene reactions, sigmatropic shifts such as hydrogen shifts, Cope and Claisen rearrangements, and the reverse. And now, just this year, Tang and Houk have verified the surmise that electrocyclic reactions also are catalyzed.^131^ This field is exploding as scientists strive to understand how enzymes frequently catalyze allowed reactions that have little or no polarity and few handles for covalent or strong noncovalent interactions. These enzymes seem to follow Pauling’s vision of enzymes:^132^ complementary to transition states, but in a noncovalent way. While in general the mechanisms of enzyme catalysis are much more complicated than this, the original Pauling model is now being manifested in pericyclases.^99,104,133−136^

### 6.8 Epilogue

Electrocyclizations were recognized as more than no-mechanism reaction puzzles—they provided Woodward and others with a desired, certain degree of stereocontrol in synthesis.^137^ Such control, when we see it, is impressive, vide the emphasis on enantiomeric excess in the hundreds of new CC coupling reactions, each better than the last. Or the thrill we feel when Frances Arnold’s group shows us how chemistry and genetic engineering can combine to evolve an enzyme that forms a C–Si bond with 90% ee for the *R* form, and a changed enzyme makes the *S* form with similar ee.^138−140^

Ultimately, the modification of pericyclic reactions blends into the broader chemical goal of reaction control. The difference here (for pericyclic reactions) is that we do not start by evolving to get control, we start by understanding the electronic transformations. We think this is important.

And why not end dreaming, with the design of a catalyst, say an enzyme with redox capabilities, that takes up the components of a forbidden cycloaddition, oxidizes one of them, reduces the other, in situ, allows the formerly forbidden, now allowed, pericyclic reaction to proceed, and then peacefully replaces the electrons in the product. Nature has already done this;^141^ can chemists do the same? What fun chemistry will be, work done and understood, the mind and hands one!

## Data Availability

The data underlying this study are available in the published article and its Supporting Information.

## Supporting Information Available

Description of computational methods, example inputs for Gaussian and ORCA, energies of stationary point structures. Final XYZ geometries for all stationary points. (PDF)
