# Supplementary material for: A 21st Century View of Allowed and Forbidden Electrocyclic Reactions
Source: J Org Chem. 2023 Dec 28;89(2):1018–34. doi: 10.1021/acs.joc.3c02103 (PMC10804416; doi:10.1021/acs.joc.3c02103)
Supplement: Supplementary file 1 — jo3c02103_si_001.pdf [file jo3c02103_si_001.pdf]

A 21st Century View of Allowed and Forbidden Electrocyclic Reactions

Qingyang Zhou[a], ‡ Garrett Kukier[a] ‡, Igor Gordiy[a]‡, Roald Hoffmann*[b], Jeffrey I. Seeman*[c], and K. N. Houk*[a]

[a] Department of Chemistry and Biochemistry, University of California, Los Angeles, California, 90095-1569, USA; E-mail: houk@chem.ucla.edu

[b] Department of Chemistry and Chemical Biology, Cornell University, Ithaca, NY 14850, United States; orcid.org/0000-0001-5369-6046; Email: rh34@cornell.edu.

[c] Department of Chemistry, University of Richmond, Richmond, Virginia 23173, USA; Email: jseeman@richmond.edu

Supporting Information

Table of Contents

A. Computational Details S1

1. Computation Methods S1
2. Example Gaussian Input File S1
3. Example ORCA Input File S2
4. Results of CASSCF(12,12)/def2-TZVP calculations S3

B. Cartesian Coordinates and Energies S4

C. References S6

D. Final geometries S8

A. Computational Details

1. Computational Methods

All the geometry optimizations and frequency calculations were carried out with the ωB97X-D[1]/6-31G(d)[2] level in Gaussian 16 program[3]. Transition structures have also been verified by intrinsic reaction coordinate (IRC)[4] calculations. For **TS-disrotatory-cyclobutene-1,3-butadiene**, IRC is calculated in Cartesian coordinates without mass-weighting to let it follow the negative frequency that leads to butadiene. CASSCF[5] and HF calculations were performed with def2-SVP[6] basis set (with def2-SVP/C[7] and def2/JK[8] as auxiliary basis sets) in ORCA 5.0.0[9-10]. Plots were generated using in-house Python scripts. The orbitals were visualized using VMD[11]. All the diradical species converge to the stable broken-symmetry open-shell singlet wavefunction.

2. Example Gaussian Input File

```
%nproc=16
%mem=16GB
#p uwb97xd/6-31g* opt=(calcfc,ts,noeigen) freq guess=mix

t

0 1
 C                  1.44795070   -0.07365659    0.18728162
 C                 -0.75102330   -0.00841059   -0.26983338
 C                  1.35699870    1.32598041   -0.34234838
 C                 -0.16327130    1.37157441   -0.62976638
 C                  1.17374070   -1.19875059   -0.73868438
 C                 -0.17518230   -1.19222459   -0.86610638
 H                  1.96718070    1.40682841   -1.24886438
 H                  1.69252770    2.08673041    0.37205662
 H                 -0.62169830    2.11840641    0.02275062
 H                 -0.37184530    1.67242341   -1.66597838
 H                  1.93429970   -1.67349559   -1.35101638
 H                 -0.75514130   -1.97391959   -1.36105338
 H                 -1.65262844   -0.02562235    0.09745891
 H                  1.74806143   -0.24640143    1.06289759
```

3. Example ORCA Input File

```
! def2-SVP def2-SVP/C def2/JK miniprint
%maxcore 1000
%pal nprocs 12 end
%casscf
trafostep RI
nel 4
norb 4
mult 1
nroots 1
end
* xyz   0   1
C     1.114138 -0.622692   0.129039
C     0.694729  0.684472  -0.093809
C    -0.694729  0.684472   0.093809
C    -1.114138 -0.622692  -0.129039
H     0.771854 -1.159058   1.005588
H     1.986814 -1.061053  -0.360181
H    -0.771854 -1.159058  -1.005588
H    -1.986814 -1.061053   0.360181
H     1.323106  1.484455  -0.478162
H    -1.323106  1.484455   0.478162
*
```

4. Results of CASSCF(12,12)/def2-TZVP calculations

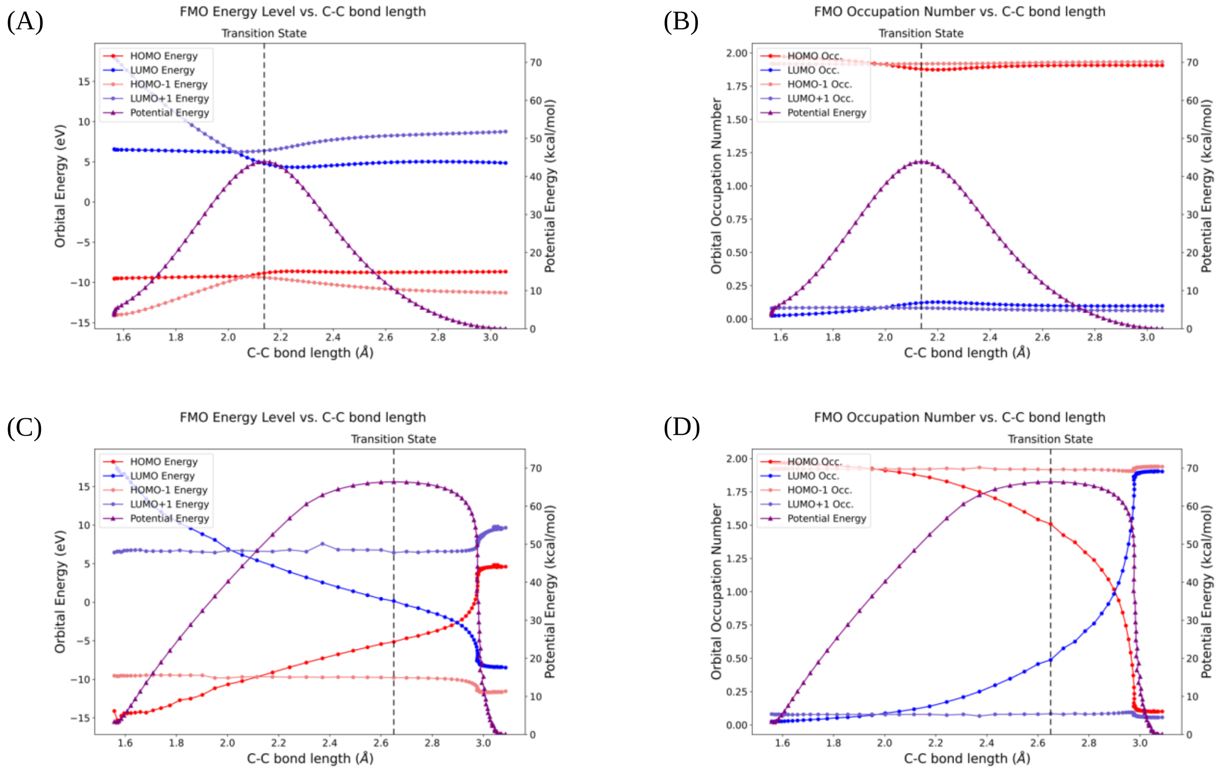

Figure S1. (A-B) Potential energy (purple) and four frontier orbital energies and FMO occupation calculated by CASSCF(12,12)/def2-TZVP along the conrotatory cyclobutene ⇋ 1,3-butadiene pathway. (C-D) Orbital energies and FMO occupation along the disrotatory cyclobutene ⇋ 1,3-butadiene pathway.

B. Cartesian Coordinates and Energies

For transition state structures, one imaginary frequency was observed and given below. For all minimum structures, no imaginary frequency was observed. Energies in hartree are reported in this section directly from the output file at the optimization level of theory (ωB97XD/6-31G(d)). The corresponding XYZ file names are provided.

Structure 2

E=-233.292427

H=-233.16429

G=-233.202769

Imag. Freq. 0

File name: structure_2.xyz

Structure 1

E=-155.927207

H=-155.835388

G=-155.866932

Imag. Freq. 0

File name: structure_1.xyz

Structures 4c/4d

E=-233.34021

H=-233.209832

G=-233.244115

Imag. Freq. 0

File names: structure_4c.xyz; structure_4d.xyz

Structure 8

E=-771.456345

H=-771.141796

G=-771.199687

Imag. Freq. 0

File name: structure_8.xyz

Structure 9c

E=-771.507014

H=-771.190592

G=-771.247302

Imag. Freq. 0

File name: structure_9c.xyz

Structure 5

E=-540.414548

H=-540.185885

G=-540.236578

Imag. Freq. 0

File name: structure_5.xyz

Structure 6

E=-540.436795

H=-540.207008

G=-540.254504

Imag. Freq. 0

File name: structure_6.xyz

TS-conrotatory-1,3,5-hexatriene-1,3-cyclohexadiene

E=-233.230825

H=-233.108208

G=-233.142901

Imag. Freq. -965.18

File name: ts_con_2_4c.xyz

TS-conrotatory-5-6

E=-540.378976

H=-540.153047

G=-540.200753

Imag. Freq. -923.98

File name: ts_con_5_6.xyz

TS-conrotatory-cyclobutene-1,3-butadiene

E=-155.857131

H=-155.767278

G=-155.796916

Imag. Freq. -784.31

File name: ts_con_1_3c.xyz

TS-disrotatory-cyclobutene-1,3-butadiene

E=-155.820551

H=-155.736241

G=-155.76821

Imag. Freq. -466.89

Imag. Freq. -464.63

File name: ts_dis_1_3d.xyz

TS-disrotatory-1,3,5-hexatriene-1,3-cyclohexadiene

E=-233.258704

H=-233.131683

G=-233.167011

Imag. Freq. -639.0

File name: ts_dis_2_4d.xyz

TS-disrotatory-8-9c

E=-771.44003

H=-771.127236

G=-771.18549

Imag. Freq. -284.1

File name: ts_dis_8_9c.xyz

Structure 3c/3d

E=-155.921212

H=-155.828617

G=-155.858293

Imag. Freq. 0

File names: structure_3c.xyz; structure_3d.xyz

References

1. Chai, J.-D.; Head-Gordon, M., Long-range Corrected Hybrid Density Functionals with Damped Atom–atom Dispersion Corrections. *Phys. Chem. Chem. Phys.* **2008**, *10*, 6615-6620.

2. Krishnan, R.; Binkley, J. S.; Seeger, R.; Pople, J. A., Self-consistent molecular orbital methods. XX. A basis set for correlated wave functions. *J. Chem. Phys.* **1980**, 72, 650– 654.

3. Gaussian 16, Revision A.03, Frisch, M. J.; Trucks, G. W.; Schlegel, H. B.; Scuseria, G. E.; Robb, M. A.; Cheeseman, J. R.; Scalmani, G.; Barone, V.; Petersson, G. A.; Nakatsuji, H.; Li, X.; Caricato, M.; Marenich, A. V.; Bloino, J.; Janesko, B. G.; Gomperts, R.; Mennucci, B.; Hratchian, H. P.; Ortiz, J. V.; Izmaylov, A. F.; Sonnenberg, J. L.; Williams-Young, D.; Ding, F.; Lipparini, F.; Egidi, F.; Goings, J.; Peng, B.; Petrone, A.; Henderson, T.; Ranasinghe, D.; Zakrzewski, V. G.; Gao, J.; Rega, N.; Zheng, G.; Liang, W.; Hada, M.; Ehara, M.; Toyota, K.; Fukuda, R.; Hasegawa, J.; Ishida, M.; Nakajima, T.; Honda, Y.; Kitao, O.; Nakai, H.; Vreven, T.; Throssell, K.; Montgomery, J. A., Jr.; Peralta, J. E.; Ogliaro, F.; Bearpark, M. J.; Heyd, J. J.; Brothers, E. N.; Kudin, K. N.; Staroverov, V. N.; Keith, T. A.; Kobayashi, R.; Normand, J.; Raghavachari, K.; Rendell, A. P.; Burant, J. C.; Iyengar, S. S.; Tomasi, J.; Cossi, M.; Millam, J. M.; Klene, M.; Adamo, C.; Cammi, R.; Ochterski, J. W.; Martin, R. L.; Morokuma, K.; Farkas, O.; Foresman, J. B.; Fox, D. J. Gaussian, Inc., Wallingford CT, 2016.

4. Fukui, K., The Path of Chemical Reactions - the IRC Approach. *Acc. Chem. Res.* **1981**, *14*, 363-368.

5. Roos, B. O.; Taylor, P. R.; Sigbahn, P. E. M. A complete active space SCF method (CASSCF) using a density matrix formulated super-CI approach. *Chem. Phys.* **1980**, *48*, 157– 173.

6. Weigend, F.; Ahlrichs, R., Balanced Basis Sets of Split Valence, Triple Zeta Valence and Quadruple Zeta Valence Quality for H to Rn: Design and assessment of accuracy. *Phys. Chem. Chem. Phys.* **2005**, *7*, 3297-3305.

7. Hellweg, A., Hättig, C., Höfener, S. *et al.* Optimized accurate auxiliary basis sets for RI-MP2 and RI-CC2 calculations for the atoms Rb to Rn. *Theor Chem Acc.* **2007***, 117*, 587–597.

8. Weigend, F. Hartree–Fock Exchange Fitting Basis Sets for H to Rn. J. Comput. Chem. 2008, 29, 167.

9. Neese, F., Software Update: the ORCA Program System, version 4.0. *Wiley Interdiscip. Rev.: Comput. Mol. Sci.* **2018**, *8*, e1327.

10. Neese, F., The ORCA Program System. *Wiley Interdiscip. Rev.: Comput. Mol. Sci.* **2012**, *2*, 73-78.

11. Humphrey, W.; Dalke, A.; Schulten, K. *J. Mol. Graphics.* **1996**, *14*, 33−38.

12. Karton, A.; Goerigk, L. Accurate reaction barrier heights of pericyclic reactions: Surprisingly large deviations for the CBS-QB3 composite method and their consequences in DFT benchmark studies. *J. Comput. Chem.* **2015**, *36* (9), 622-632.

13. Karton, A.; Tarnopolsky, A.; Lamère, J.-F.; Schatz, G. C.; Martin, J. M. L. Highly Accurate First-Principles Benchmark Data Sets for the Parametrization and Validation of Density Functional and Other Approximate Methods. Derivation of a Robust, Generally Applicable, Double-Hybrid Functional for Thermochemistry and Thermochemical Kinetics. *J. Phys. Chem. A* **2008**, *112* (50), 12868-12886.

14. Guner, V.; Khuong, K. S.; Leach, A. G.; Lee, P. S.; Bartberger, M. D.; Houk, K. N. A Standard Set of Pericyclic Reactions of Hydrocarbons for the Benchmarking of Computational Methods: The Performance of ab Initio, Density Functional, CASSCF, CASPT2, and CBS-QB3 Methods for the Prediction of Activation Barriers, Reaction Energetics, and Transition State Geometries. *J. Phys. Chem. A* **2003**, *107* (51), 11445-11459.

15. Hauser, W. P.; Walters, W. THE KINETICS OF THE THERMAL ISOMERIZATION OF CYCLOBUTENE1, 2. *The Journal of Physical Chemistry* **1963**, *67* (6), 1328-1333.

1_3_5_hexatriene

14

```
H     0.691249 -1.725431  -0.409068
C     1.564638 -1.250124   0.017742
C     0.725448  1.135085  -0.203981
C    -1.551457  0.144847   0.369317
C    -0.612038  1.182703  -0.085498
C    -1.783745 -1.024784  -0.225834
C     1.681428  0.074256   0.140629
H    -1.079871  2.146890  -0.289551
H    -1.270839 -1.310777  -1.139505
H     2.622565  0.458027   0.536777
H     1.203020  2.065437  -0.511102
H    -2.156232  0.416537   1.236158
H    -2.524775 -1.718453   0.160486
H     2.369236 -1.904130   0.341552
```

####################################

1_3_butadiene

10

```
C    -0.280546  1.508631  -0.491925
C    -0.280546  0.680155   0.554109
C     0.280546 -0.680155   0.554109
C     0.280546 -1.508631  -0.491925
H     0.180665  1.232023  -1.436697
H    -0.729433  2.495378  -0.433094
H    -0.180665 -1.232023  -1.436697
H     0.729433 -2.495378  -0.433094
H    -0.706983  1.021761   1.496685
H     0.706983 -1.021761   1.496685
```

####################################

1_3_cyclohexadiene

14

```
H     1.343325 -0.744375   1.337910
C     1.191250 -0.725764   0.246808
C    -1.255625 -0.726562  -0.107308
C    -0.112677  1.420227   0.063298
C    -1.256036  0.725839   0.107292
C     1.190818  0.726447  -0.246803
C    -0.111874 -1.420280  -0.063284
H    -2.204504  1.226157   0.283419
H     2.035116  1.269513   0.190542
H    -0.111172 -2.499909  -0.190011
H    -2.203841 -1.227362  -0.283420
H    -0.112659  2.499840   0.190147
```

H	1.342799	0.745030	-1.337929
H	2.035797	-1.268331	-0.190678

####################################

cyclobutene

10

C	0.000131	0.782737	-0.698148
C	-0.000131	0.668126	0.812545
C	0.000131	-0.668126	0.812545
C	-0.000131	-0.782737	-0.698148
H	0.890821	1.241234	-1.141926
H	-0.889888	1.241838	-1.142632
H	-0.890821	-1.241234	-1.141926
H	0.889888	-1.241838	-1.142632
H	-0.000318	1.417796	1.598178
H	0.000318	-1.417796	1.598100

####################################

structure_3

14

C	0.078480	0.784094	0.565617
C	0.078450	-0.784148	0.565541
C	-1.230361	0.774903	-0.281976
C	-1.230555	-0.774856	-0.281756
C	1.349838	0.669760	-0.241886
C	1.349781	-0.669906	-0.241938
H	-1.154279	1.261988	-1.257802
H	-2.085018	1.199268	0.251817
H	-2.085131	-1.198893	0.252409
H	-1.154779	-1.262152	-1.257502
H	1.969923	1.417389	-0.729598
H	1.969762	-1.417567	-0.729730
H	0.082806	-1.374228	1.484520
H	0.082914	1.375112	1.484276

####################################

structure_4

14

C	0.392963	-1.103228	0.496222
C	-0.725483	1.115591	0.038445
C	1.474582	-0.343283	-0.189980
C	0.803828	1.103762	-0.043210
C	-0.757663	-1.189965	-0.223861
C	-1.542629	0.032836	0.024295
H	1.564926	-0.600106	-1.250355
H	2.467541	-0.346415	0.271993

H 1.187909 1.536771 0.889327
H 1.131145 1.779746 -0.846060
H -0.709354 -1.514723 -1.266599
H -2.626085 0.139769 -0.008791
H -1.166067 2.113818 0.057929
H 0.276399 -0.803143 1.541087

###################################

structure_5

26

C 2.957913 -1.667559 0.194922
C 3.764804 -0.727879 -0.342659
C 3.509746 0.691237 -0.447141
C 1.657354 -1.468660 0.794546
C 2.365931 1.379033 -0.244132
C 0.844704 -0.391180 0.758540
C 1.026962 0.907910 0.113475
H 3.332116 -2.688345 0.222693
H 4.733679 -1.054816 -0.712654
H 4.367016 1.284600 -0.758286
H 1.274031 -2.326921 1.342337
H 2.425098 2.454106 -0.407721
H -0.102423 -0.494354 1.281693
C -0.007163 1.788676 -0.033270
C -1.416801 1.603769 0.240675
H 0.257339 2.800216 -0.340364
C -2.208638 0.526781 0.010217
C -3.627053 0.447767 0.383730
C -1.901251 -0.727796 -0.686011
C -4.106385 -0.745020 -0.032039
C -3.028263 -1.474721 -0.709499
H -5.116386 -1.115763 0.097204
H -3.139726 -2.449922 -1.169432
H -1.916215 2.485336 0.646596
H -4.160744 1.224669 0.917770
H -0.944953 -0.972956 -1.127958

###################################

structure_6

26

C 2.331988 -1.729558 0.185672
C 3.366230 -0.776894 -0.187434
C 3.248753 0.557963 -0.305976
C 1.039343 -1.524284 0.470071
C 2.068934 1.385467 -0.137345
C 0.176309 -0.289592 0.483343
C 0.787655 1.075296 0.161982
H 2.677276 -2.758504 0.270221
H 4.348344 -1.207097 -0.369621

H	4.146191	1.108055	-0.580518
H	0.477093	-2.407501	0.770991
H	2.254647	2.444356	-0.314972
H	-0.234579	-0.210865	1.504352
C	-0.182853	2.177298	0.183600
C	-1.514401	1.990952	0.073709
H	0.213497	3.185008	0.283612
C	-2.038182	0.660744	-0.132639
C	-3.276799	0.145080	0.034870
C	-1.069402	-0.453672	-0.429156
C	-3.201847	-1.310649	-0.087088
C	-1.927049	-1.683231	-0.313354
H	-4.056079	-1.974667	-0.011997
H	-1.575312	-2.691936	-0.492394
H	-2.199590	2.831582	0.145140
H	-4.177677	0.702900	0.267028
H	-0.725895	-0.370845	-1.473378

####################################

structure_8

17

C	0.819635	0.490428	-0.058290
C	-0.451178	-0.480985	0.173151
C	1.344218	-0.509799	-1.124526
C	0.138562	-1.452872	-0.895388
C	1.179514	0.130459	1.363879
C	0.122677	-0.663491	1.561813
H	2.338539	-0.917514	-0.928425
H	1.343741	-0.065463	-2.124523
H	-0.503247	-1.556041	-1.772679
H	0.368940	-2.446772	-0.506149
H	2.061404	0.365448	1.952544
H	-0.181089	-1.324361	2.366975
C	-1.794411	0.014758	0.002564
N	-2.847894	0.470596	-0.164703
N	0.809688	1.863252	-0.418235
H	0.320478	2.424651	0.272327
H	0.364573	2.012126	-1.318721

####################################

structure_9

17

C	-1.721546	-0.108675	0.023087
C	1.129770	-0.072783	-0.052032
C	-1.004745	1.195729	0.286295
C	0.391649	1.218830	-0.336950
C	-1.008866	-1.259449	-0.073165
C	0.431113	-1.227789	0.027185
H	-0.930067	1.320973	1.376929

H	-1.594273	2.036375	-0.095486
H	0.304764	1.342271	-1.426531
H	0.950457	2.080617	0.037856
H	-1.510797	-2.216862	-0.176571
H	0.959156	-2.166970	0.165206
C	2.551763	-0.058148	0.037846
N	3.710874	-0.015903	0.110703
N	-3.087709	-0.039955	-0.067657
H	-3.610281	-0.899454	0.017557
H	-3.545942	0.767758	0.326114

####################################

structure_10

36

C	4.090570	-0.702996	-0.740905
C	2.956278	-1.377046	-0.362254
C	1.743277	-0.718518	0.001584
C	1.743249	0.718548	0.001484
C	2.956203	1.377068	-0.362523
C	4.090526	0.703004	-0.741057
C	0.658377	-1.540152	0.428128
C	-0.757145	-1.289759	0.179440
C	-1.396204	-0.000007	0.000635
C	-0.757183	1.289763	0.179494
C	0.658354	1.540195	0.428042
C	-1.544574	-2.438795	0.055854
C	-2.907331	-2.411986	-0.257284
C	-3.527130	-1.209673	-0.433262
C	-2.800155	-0.000028	-0.291976
C	-3.527192	1.209596	-0.433136
C	-2.907448	2.411919	-0.257046
C	-1.544679	2.438769	0.056040
C	1.023161	2.873375	1.047145
C	1.023146	-2.873293	1.047335
H	4.974049	-1.254453	-1.047736
H	2.968500	-2.460536	-0.390801
H	2.968367	2.460552	-0.391287
H	4.973969	1.254449	-1.048017
H	-1.082323	-3.410910	0.162360
H	-3.454126	-3.344510	-0.359428
H	-4.585707	-1.152979	-0.670601
H	-4.585768	1.152873	-0.670468
H	-3.454291	3.344427	-0.359083
H	-1.082481	3.410904	0.162587
H	0.277704	3.164797	1.792209
H	1.986326	2.806892	1.557770
H	1.093614	3.694698	0.318723
H	0.277587	-3.164714	1.792297
H	1.093735	-3.694642	0.318956
H	1.986238	-2.806754	1.558092

####################################

structure_11c

36

C	-3.536842	0.699332	-1.605673
C	-2.643698	1.435303	-0.817749
C	-1.771943	0.693055	-0.041969
C	-1.771928	-0.693070	-0.041986
C	-2.643664	-1.435316	-0.817788
C	-3.536825	-0.699346	-1.605693
C	-0.627909	0.808014	0.963912
C	0.705801	1.177734	0.348237
C	1.407097	0.000003	0.013900
C	0.705817	-1.177734	0.348246
C	-0.627901	-0.808025	0.963901
C	1.281010	2.391808	0.078123
C	2.564192	2.421101	-0.532272
C	3.246922	1.271896	-0.861326
C	2.673838	0.000010	-0.592866
C	3.246939	-1.271871	-0.861315
C	2.564226	-2.421083	-0.532252
C	1.281043	-2.391803	0.078142
C	-0.937633	-1.533617	2.264776
C	-0.937625	1.533608	2.264800
H	-4.248807	1.224219	-2.236501
H	-2.650218	2.521408	-0.829657
H	-2.650157	-2.521421	-0.829727
H	-4.248777	-1.224232	-2.236534
H	0.778509	3.325258	0.317329
H	3.017634	3.385446	-0.743900
H	4.228019	1.332850	-1.324954
H	4.228037	-1.332815	-1.324944
H	3.017683	-3.385423	-0.743872
H	0.778555	-3.325259	0.317354
H	-0.149574	-1.360723	3.005172
H	-1.893764	-1.204461	2.683205
H	-1.004671	-2.613282	2.089835
H	-0.149528	1.360762	3.005165
H	-1.004716	2.613273	2.089862
H	-1.893722	1.204416	2.683283

####################################

ts_con_1_3_5_hexatriene_1_3_cyclohexadiene

14

C	0.424795	1.085386	-1.136092
C	-0.224580	1.467680	0.071180
C	-0.224580	0.679174	1.217554
C	0.224580	-0.679174	1.217554
C	0.224580	-1.467680	0.071180
C	-0.424795	-1.085386	-1.136092
H	1.386778	0.585831	-1.095949

H 0.208991 1.624575 -2.054301
H -1.386778 -0.585831 -1.095949
H -0.208991 -1.624575 -2.054301
H -0.788446 2.399373 0.082739
H -0.576950 1.111265 2.151663
H 0.576950 -1.111265 2.151663
H 0.788446 -2.399373 0.082739

####################################

ts_dis_1_3_5_hexatriene_1_3_cyclohexadiene

14

H -0.930774 -1.489164 1.079887
C -1.136674 -1.200628 0.062147
C -0.698975 1.232954 0.162640
C 1.473193 0.123046 -0.194133
C 0.699779 1.232606 0.162450
C 1.135904 -1.201184 0.062358
C -1.473109 0.123890 -0.194017
H 1.172933 2.211848 0.115877
H 0.929729 -1.489276 1.080173
H -2.309783 0.326591 -0.863964
H -1.171670 2.212435 0.116303
H 2.310152 0.325130 -0.863902
H 1.629381 -1.981354 -0.516311
H -1.630671 -1.980309 -0.516743

####################################

ts_con_cyclobutene_1_3_butadiene

10

C 0.102167 1.063571 -0.620573
C -0.102167 0.681882 0.728337
C 0.102167 -0.681882 0.728337
C -0.102167 -1.063571 -0.620573
H 1.060136 0.862202 -1.084793
H -0.452159 1.881057 -1.092493
H -1.060136 -0.862202 -1.084793
H 0.452159 -1.881057 -1.092493
H -0.450165 1.328103 1.530697
H 0.450165 -1.328103 1.530697

####################################

ts_dis_cyclobutene_1_3_butadiene

10

C -0.003551 -0.617797 -1.324943
C -0.003550 0.702021 -0.666637
H -0.853147 -0.943916 -1.919660

```
H     0.918818 -1.183214  -1.432590
H    -0.023060  1.621790  -1.254478
C    -0.003550  0.702021   0.666637
C    -0.003551 -0.617797   1.324943
H    -0.023060  1.621790   1.254478
H    -0.853147 -0.943916   1.919660
H     0.918818 -1.183214   1.432590
```

####################################

ts_dis_3_1_3_cyclohexadiene

14

```
C     0.085258 -1.114477  -0.475260
C     0.084987  1.114419  -0.475388
C    -1.219477 -0.772130   0.228593
C    -1.219608  0.772045   0.228612
C     1.331009 -0.667951   0.194997
C     1.330817  0.668316   0.195090
H    -1.274758 -1.210755   1.235267
H    -2.070080 -1.158819  -0.340392
H    -2.070305  1.158654  -0.340284
H    -1.274878  1.210548   1.235331
H     2.061123 -1.329660   0.659315
H     2.060671  1.330183   0.659584
H     0.104933  1.882923  -1.245045
H     0.105377 -1.884410  -1.243646
```

####################################

ts_con_3_4

14

```
C     0.121581 -0.900721   0.587243
C    -0.410519  1.114186   0.216992
C     1.395299 -0.636035  -0.151724
C     1.065292  0.888418  -0.240700
C    -1.065467 -0.993421  -0.179928
C    -1.515154  0.324871  -0.147228
H     1.401326 -1.116096  -1.134216
H     2.332739 -0.844099   0.371465
H     1.697775  1.396566   0.493710
H     1.266833  1.353630  -1.217298
H    -1.244841 -1.739151  -0.950700
H    -2.512261  0.718135  -0.356445
H    -0.571663  2.112345   0.637359
H     0.083902 -0.665113   1.648199
```

####################################

ts_con_4_5

26

C	-2.328727	-1.702195	-0.351332
C	-3.286400	-0.854631	0.222505
C	-3.166733	0.510311	0.423628
C	-1.056861	-1.402511	-0.798074
C	-2.076736	1.361531	0.215180
C	-0.313445	-0.207665	-0.679205
C	-0.774537	1.084392	-0.214154
H	-2.649853	-2.729403	-0.514135
H	-4.238312	-1.305817	0.487727
H	-4.050854	1.001835	0.825401
H	-0.522583	-2.207293	-1.295119
H	-2.250683	2.401412	0.483829
H	0.565784	-0.168321	-1.313029
C	0.193499	2.154406	-0.246846
C	1.538064	1.925698	-0.117592
H	-0.180385	3.168996	-0.359628
C	2.056942	0.658453	0.268019
C	3.148842	0.001891	-0.337808
C	1.283029	-0.347414	0.962175
C	3.057083	-1.368153	-0.048693
C	1.939611	-1.576650	0.777187
H	3.745410	-2.132658	-0.389981
H	1.659176	-2.517122	1.237496
H	2.230363	2.738906	-0.334391
H	3.867753	0.467186	-1.003373
H	0.542397	-0.142498	1.725274

####################################

ts_dis_7_8

17

C	1.389189	-0.095370	0.198029
C	-0.809539	-0.030088	-0.259237
C	1.298706	1.304020	-0.332298
C	-0.221682	1.349918	-0.619030
C	1.115062	-1.220713	-0.727633
C	-0.233844	-1.214078	-0.855273
H	1.908421	1.384038	-1.239196
H	1.634906	2.065106	0.381426
H	-0.679636	2.096502	0.034106
H	-0.430816	1.651307	-1.654977
H	1.875584	-1.695048	-1.340355
H	-0.813847	-1.995699	-1.350290
C	-2.117007	-0.054812	0.273189
N	-3.184522	-0.069639	0.746275
N	1.809746	-0.337368	1.427291
H	1.772191	-1.289819	1.767529
H	1.831322	0.399392	2.120315

####################################

ts_dis_9_10

36

C	3.648844	-0.696609	-1.451466
C	2.691310	-1.406014	-0.723363
C	1.741283	-0.694346	0.000317
C	1.741281	0.694343	0.000304
C	2.691311	1.405998	-0.723385
C	3.648845	0.696580	-1.451477
C	0.655872	-1.231234	0.870757
C	-0.710522	-1.237655	0.326698
C	-1.343139	-0.000002	-0.010211
C	-0.710527	1.237656	0.326694
C	0.655872	1.231250	0.870737
C	-1.407340	-2.415287	0.133696
C	-2.696464	-2.417790	-0.440630
C	-3.302156	-1.239161	-0.790786
C	-2.649680	-0.000005	-0.559075
C	-3.302166	1.239146	-0.790780
C	-2.696482	2.417779	-0.440620
C	-1.407356	2.415283	0.133699
C	1.031041	2.212583	1.941069
C	1.031018	-2.212528	1.941132
H	4.404645	-1.235017	-2.015940
H	2.699805	-2.492781	-0.714353
H	2.699808	2.492765	-0.714395
H	4.404646	1.234979	-2.015959
H	-0.946237	-3.361951	0.401795
H	-3.207025	-3.363023	-0.599727
H	-4.301472	-1.235538	-1.218144
H	-4.301483	1.235517	-1.218136
H	-3.207051	3.363009	-0.599712
H	-0.946256	3.361949	0.401796
H	0.206233	2.378191	2.640401
H	1.902943	1.855661	2.499501
H	1.308654	3.191473	1.516445
H	0.206284	-2.377897	2.640609
H	1.308358	-3.191523	1.516569
H	1.903078	-1.855724	2.499389
